# Middle Pleistocene re-organization of Australian Monsoon

**DOI:** 10.1038/s41467-023-37639-x

**Published:** 2023-04-10

**Authors:** Li Gong, Ann Holbourn, Wolfgang Kuhnt, Bradley Opdyke, Yan Zhang, Ana Christina Ravelo, Peng Zhang, Jian Xu, Kenji Matsuzaki, Ivano Aiello, Sebastian Beil, Nils Andersen

**Affiliations:** 1 Institute of Geosciences, Christian-Albrechts-University, D-24118 Kiel, Germany; 2 Research School of Earth Sciences, Australian National University, Mills Road, Acton, ACT 2601 Australia; 3 Ocean Sciences Department, University of California, 1156 High Street, Santa Cruz, CA 95064 USA; 4 Institute of Cenozoic Geology and Environment, State Key Laboratory of Continental Dynamics and Department of Geology, Northwest University, 710069 Xi’an, China; 5 Atmosphere and Ocean Research Institute, The University of Tokyo, 5-1-5, Kashiwanoha, Kashiwa, Chiba, 277-8564 Japan; 6 Department of Earth and Planetary Science, Graduate School of Science, The University of Tokyo, 7-3-1, Hongo, Bunkyo-ku, Tokyo, 113-0033 Japan; 7 Department of Geological Oceanography, Moss Landing Marine Laboratories, San Jose State University, Moss Landing, CA 95039 USA; 8 Leibniz Laboratory for Radiometric Dating and Stable Isotope Research, Christian-Albrechts-University Kiel, D-24118 Kiel, Germany

**Keywords:** Palaeoclimate, Palaeoceanography

## Abstract

The sensitivity of the Australian Monsoon to changing climate boundary conditions remains controversial due to limited understanding of forcing processes and past variability. Here, we reconstruct austral summer monsoonal discharge and wind-driven winter productivity across the Middle Pleistocene Transition (MPT) in a sediment sequence drilled off NW Australia. We show that monsoonal precipitation and runoff primarily responded to precessional insolation forcing until ~0.95 Ma, but exhibited heightened sensitivity to ice volume and *p*CO_2_ related feedbacks following intensification of glacial-interglacial cycles. Our records further suggest that summer monsoon variability at the precessional band was closely tied to the thermal evolution of the Indo-Pacific Warm Pool and strength of the Walker circulation over the past ~1.6 Myr. By contrast, productivity proxy records consistently tracked glacial-interglacial variability, reflecting changing rhythms in polar ice fluctuations and Hadley circulation strength. We conclude that the Australian Monsoon underwent a major re-organization across the MPT and that extratropical feedbacks were instrumental in driving short- and long-term variability.

## Abstract (web summary)

The response of monsoons to climate change remains uncertain. Here, the authors show that the Australian Summer Monsoon was primarily driven by insolation forcing but exhibited high sensitivity to ice volume and *p*CO_2_ after ~0.95 Ma. By contrast, wind-driven winter productivity tracked glacial-interglacial variability over the past 1.6 Myr.

## Introduction

Monsoons exert a fundamental influence on the planetary transport of energy and water vapor through the atmosphere and are a central component of the climate system^1^. To date, there is still considerable debate concerning the future development of monsoonal systems and associated changes in regional rainfall^2,3^. Some recent modeling projections indicated that monsoonal precipitation will increase with rising atmospheric *p*CO_2_ and global warming due to enhanced moisture convergence from surface evaporation, despite a partial offset from a weaker atmospheric circulation^4,5^. However, regional monsoons exhibit different responses to external insolation forcing and internal boundary conditions, although they are intrinsically linked through oceanic and atmospheric processes controlling the meridional and zonal exchange of energy and moisture^6,7^. Based on model simulations and proxy-based paleoclimatic reconstructions, a more dynamic view of regional convective monsoonal rainfall has emerged, which contrasts with earlier perceptions of monsoons as large-scale sea breeze circulation, driven by the land-sea temperature contrast^8,9^.

The unique setting of the Australian Summer Monsoon (ASM) at the southern edge of the largest amplitude seasonal swing of the Intertropical Convergence Zone (ITCZ) within the large-scale Austral–Asian Monsoon system makes it a highly sensitive monitor of tropical hydroclimate variability. The ASM is less susceptible to local orographic rainfall than its Northern Hemisphere (NH) counterparts, as the lack of high mountain chains in this region enables direct moisture transport from the source regions to the core area of monsoon precipitation. In addition, the discharge of the monsoonal terrigenous sediment load to the adjacent coastal margin is directly routed through a few and relatively short rivers and does not flow through an extensive network of flood plains and delta systems. However, the sensitivity of the ASM to changing climate boundary conditions such as ice volume and greenhouse gas concentrations and the inter-hemispheric coupling with other monsoonal subsystems remain matters of intense debate. This lack of consensus concerning the ASM is largely due to our limited understanding of past variability and conflicting results from model simulations^7,10^.

**Fig. 1:**
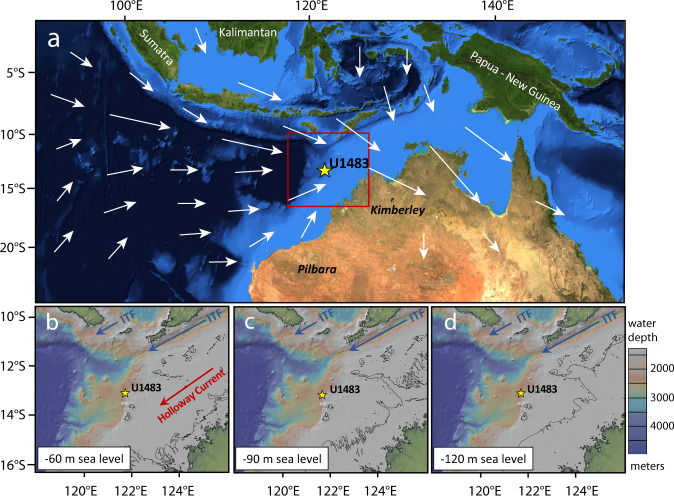
Present-day monsoonal circulation in Eastern Indian Ocean and glacial coastlines and currents. **a** Location of IODP Site U1483 in the Timor Sea off NW Australia. Satellite image and bathymetry from Blue Marble: Next Generation (BMNG)^116^, courtesy of NASA Earth Observatory (NASA/JPL-Caltech). White arrows indicate prevalent wind patterns associated with the Australian Summer Monsoon^29^. **b**–**d** Modern bathymetry and coastline position at sea level 60, 90, and 120 m below present. Main pathways of the cool thermocline-dominated Indonesian Throughflow (ITF) and the warm surface Holloway Current are indicated by blue and red arrows. Note that the pathway of the Holloway Current is blocked during low eustatic sea level. Base maps generated with GeoMapApp (www.geomapapp.org)/CC BY using the Global Multi-Resolution Topography (GMRT) synthesis^117^.

Today, the ASM delivers ~85% of the annual rainfall to northern Australia (north of 25°S) during the austral summer season^11,12^. Intensified and prolonged rainfall during January-March massively increases the load transport of major rivers including the Fitzroy and Ord Rivers (Fig. 1), leading to enhanced terrigenous deposition along the NW Australian continental margin^13^. The annual sediment discharge of the Ord River was evaluated to be in the order 34 Mt/yr (prior to dam construction)^14^, while the discharge of the Fitzroy River was estimated to be slightly higher^15^. The periodic increases in monsoonal precipitation and terrigenous discharge in northern Australia are associated with the seasonal displacement of the ITCZ, which migrates south during austral summer, reaching the Kimberley (~15°S) and northern Pilbara (~20°S) in February, when the peak of the wet season occurs^16^. In contrast, vast areas of northern Australia dry out, and Trade winds carry increased amounts of dust from the continent into the eastern Indian Ocean, when the ITCZ is locked into a more northerly position during austral winter (July–September), and the dry Australian Winter Monsoon (AWM) prevails.

Peak productivity occurs during austral winter, when cooler sea surface temperatures (SSTs) and intensified Trade winds promote convective mixing of the upper water column and shoaling of the nutricline and chlorophyll maximum layer off NW Australia^17,18^. Chlorophyll concentrations reach 0.2–0.4 mg/m^3^ during austral winter (August), dropping to 0.08–0.1 mg/m^3^ during austral summer (January)^19^. The westward flow of warm, low-density surface waters from the Indonesian Archipelago through the Timor Passage (Indonesian Throughflow) and across the NW Australian Shelf (Holloway Current) (Fig. 1) additionally affects the seasonal and interannual variability of upper ocean mixing and productivity in the Timor Sea^20–22^. During the ASM season, westerly winds pile up warm, fresh and nutrient-depleted surface water masses over shelf areas, forming a freshwater cap that inhibits productivity^23^. When monsoonal winds reverse in austral fall, this freshwater cap disappears and convective mixing increases, as tropical surface water masses flow southwestwards along the coast, driven by a strong NE-SW sea-level gradient, thus forming the Holloway Current^21,24^. Previous studies of late Pleistocene glacial–interglacial productivity in this region showed that the accumulation of chlorophyll alpha, chlorins and marine organic matter is decoupled from that of pelagic carbonate^25,26^. The organic export flux records display a distinct maximum during glacial stages, whereas carbonate accumulation rates exhibit little glacial–interglacial variability.

Proxy-based investigations of past Australian climate change in relation to secular shifts in the latitudinal displacement of the ITCZ and the variability of the Walker circulation have primarily focused on the last few glacial cycles^27–32^. These studies indicated that changes in the intensity of the ASM and the latitudinal migration of the ITCZ are linked to precessional insolation, but are also strongly influenced by mean-state background conditions such as high-latitude ice cover, greenhouse gas concentrations and the inter-hemispheric thermal gradient. However, the role of external (insolation) forcing versus internal feedback in driving monsoonal climate variability remains highly enigmatic. The hypothesis that changing atmospheric *p*CO_2_ levels, global temperatures and ice volume exert a major control on the ASM development needs to be tested, in particular, for warmer periods with different boundary conditions than today and prior to the emergence of late Pleistocene high-amplitude glacial–interglacial cycles.

Within the long-term Neogene cooling trend, the Middle Pleistocene Transition (MPT, ~1.2–0.8 Ma) stands out as a period of special interest, as it marks a major shift in the response of Earth’s climate to orbital forcing. During this period, Earth’s climate transitioned from an overall warmer phase with a dominant 41 kyr periodicity and reduced inter-hemispheric thermal contrast to a high-amplitude, quasi-periodic (~100 kyr) mode of glacial–interglacial variability^33–36^. This transition occurred in the absence of substantial changes in orbital forcing, indicating that it was mainly driven by internal feedback within the coupled ocean-climate system. To date, the driving mechanisms of this climate shift and the evolving relationships of high- and low-latitude climate change remain unclear^36^. This interval, therefore, offers a challenging opportunity to explore monsoon dynamics under different mean-states of Earth’s climate variability and to investigate tropical climate evolution in relation to major changes in high-latitude climate, atmospheric *p*CO_2_ and the latitudinal temperature gradient, thus, helping to constrain projections of climate change and sensitivity.

During International Ocean Discovery Program (IODP) Expedition 363 “Western Pacific Warm Pool”, an undisturbed Pleistocene hemipelagic succession was retrieved at Site U1483 (13°5.24’S, 121°48.25’E; 1733 m water depth) in the Timor Sea off NW Australia^37^, at the southwestern edge of the present-day Indo-Pacific Warm Pool (IPWP). This carbonate- and clay-rich sequence, which exhibits cyclic variations on decimeter to meter scales, provides an ideal archive to document the evolution of the wet (ASM) and dry (AWM) phases of NW Australian climate under different climate boundary conditions prior to and following the MPT. In this work, we integrate high-resolution benthic foraminiferal δ^18^O data, X-ray fluorescence (XRF) scanner-derived elemental data, light reflectance spectroscopy and spectral gamma ray records to monitor secular variations in terrigenous river discharge, productivity, and bottom water oxygenation and to investigate the primary drivers of monsoonal climate evolution between 1.6 and 0.4 Ma. This work extends published isotope and XRF-scanner data records (0.4–0 Ma) from the same site^31^, providing a continuous record of summer and winter monsoon variability spanning the past 1.6 Myr.

## Results and discussion

### Temporal evolution of benthic foraminiferal δ^18^O

**Fig. 2:**
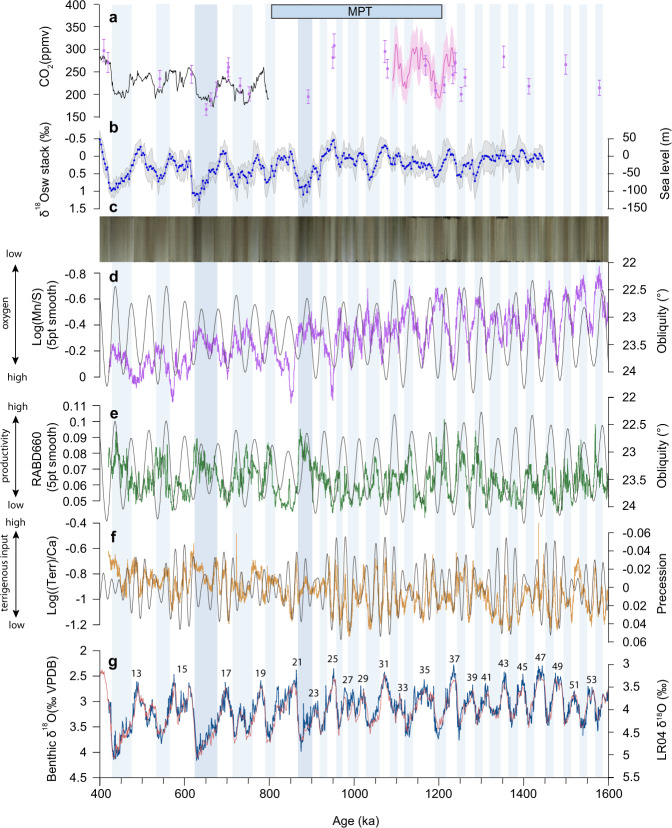
Comparison of Australian Summer and Winter Monsoon proxy records with atmospheric *p*CO_2_ and sea-level reconstructions between 1.6 and 0.4 Ma. **a** Atmospheric *p*CO_2_, black: ice core composite record^118^, pink: δ^11^B-based data across the middle Pleistocene Transition (MPT) (with 2σ error envelope^73^), purple: δ^11^B record^71^ (with 2σ error). **b** Sea level reconstruction based on seawater δ^18^O (δ^18^O_sw_) stack (with 1σ error envelope) from ref. ^67^. **c** U1483 composite core linescan image. **d** U1483 Log(Mn/S) 5 point moving average (purple) with obliquity (gray). Long-term increasing trend after ~1 Ma related to compaction and associated changes in pore water content in upper part of sediment succession (Supplementary Material 2). **e** U1483 RABD_660_ 5 point moving average (green) with obliquity (gray). **f** U1483 Log(Terr/Ca) (brown) with precession (gray). **g** U1483 benthic foraminiferal δ^18^O (blue) with LR04 δ^18^O stack (red)^38^; numbering refers to Marine Isotope Stages (MIS). Glacial intervals shaded light blue; marked increases in ice volume during MIS 22 and 16 shaded darker blue.

Our high-resolution (~1 kyr) benthic foraminiferal δ^18^O record from Site U1483 extends from ~1.6 to 0.4 Ma, encompassing Marine Isotope Stage (MIS) 55 to MIS12 (Fig. 2). The δ^18^O series exhibits similar structure and trends as the LR04 oxygen isotope stack^38^, which was used as a correlation target to generate the Site U1483 age model between ~1.6 and 0.4 Ma (Supplementary Table S1 and Supplementary Fig. S1). A distinctive feature of the benthic foraminiferal δ^18^O record is the shift from dominant 41 kyr periodicity to a mix of ~100 and 41 kyr variability after 0.95 Ma (Fig. 2 and Supplementary Fig. S1). Sedimentation rates average 10 cm/kyr between 420.7 and 1599.8 ka, with a maximum of 16.8 cm/kyr and a minimum of 5.6 cm/kyr, and with no apparent long-term trend (Supplementary Fig. S1).

### Variations in wind-driven primary productivity and bottom water oxygenation

**Fig. 3:**
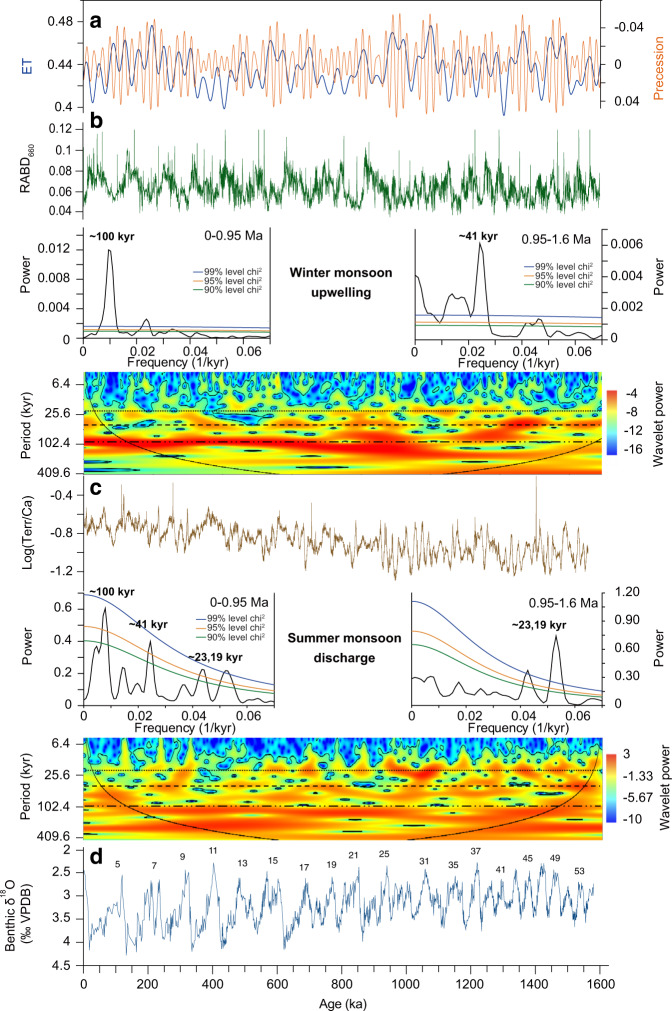
Spectral characteristics of Australian Summer and Winter Monsoon proxy records across the Middle Pleistocene Transition. **a** Eccentricity and obliquity (ET), precession parameter from ref. ^119^. **b** U1483 reflectance spectroscopy RABD_660_ data. **c** U1483 XRF-scanner-derived Log(Terr/Ca). **d** U1483 benthic foraminiferal δ^18^O; data from 0 to 410 ka from ref. ^31^. Confidence intervals of 99%, 95%, and 90% are given as blue, orange, and green lines. Continuous wavelet power spectrum computed with Morlet basis function in Past4.04^114^; black line indicates cone of influence; dotted lines indicate 23, 41, 100 kyr periods.

**Fig. 4:**
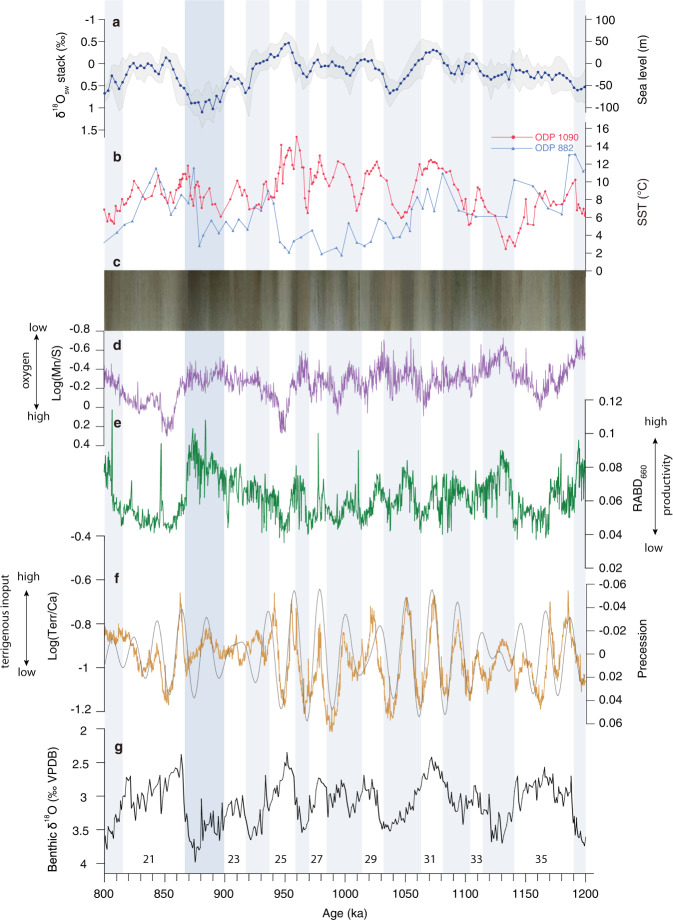
Evolution of NW Australian climate across the Middle Pleistocene Transition. **a** Sea-level reconstruction based on seawater δ^18^O (δ^18^O_sw_) stack (with 1σ error envelope) from ref. ^67^. **b** High-latitude NH and SH sea surface temperature (SST) records, red: SST record from ODP Site 1090 in subantarctic Atlantic Ocean^47^ (42°54.8’S, 8°53.9’E), blue: SST record from ODP Site 882 in subarctic Pacific Ocean^47^ (50°21’N, 167°35’W). **c** U1483 composite core linescan image; **d** U1483 Log(Mn/S). **e** U1483 RABD_660_. **f** U1483 Log(Terr/Ca) is highly coherent with precession (gray). **g** U1483 benthic foraminiferal δ^18^O; numbering refers to Marine Isotope Stages (MIS). Glacial intervals shaded light blue; marked increase in ice volume during MIS 22 shaded darker blue.

We use visible light relative absorbance band depth at 660 nm (RABD_660_ in ~250 yr resolution) derived from color reflectance spectroscopy^37^, XRF-scanner-derived logarithmic ratio of manganese and sulfur (Log(Mn/S) in ~200 yr resolution) and spectral gamma ray-derived uranium (U) concentrations^39^ (~1 kyr resolution) to monitor variations in primary productivity and in bottom water oxygenation at Site U1483 (Fig. 2 and Material and Methods, Supplementary Material 2). Proxy indicators of primary productivity (RABD_660_) and bottom water oxygenation (Log(Mn/S) and U concentrations) mostly exhibit spectral signals similar to that of global ice volume over the past 1.6 Myr (Figs. 2 and 3 and Supplementary Figs. S2–S5). A 41 kyr obliquity signal dominates prior to ~1.2 Ma in the REDFIT and wavelet power spectra, which is replaced by a mix of 41 and 100 kyr variability after ~0.95 Ma (Supplementary Figs. S4 and S5). The AWM proxy records RABD_660_ and Log(Mn/S) display a distinct precessional signal between ~1.1 and ~0.95 Ma during an interval, which is also characterized by high-amplitude variance in orbital precession (Fig. 3 and Supplementary Figs. S4 and S5). The AWM switch to 41 and 100 kyr variability after ~0.95 Ma was approximately coeval with a relatively abrupt change in the spectral pattern of the ASM proxy records from dominant precessional periodicity to mixed 100, 41, 23, and 19 kyr variability (Figs. 2–4).

### Variations in monsoonal terrigenous discharge

We use XRF-scanner-derived elemental signatures (~200 yr time resolution) to evaluate temporal changes in the terrigenous discharge from the adjacent Ord and Fitzroy Rivers in NW Australia and precipitation changes over the catchment areas of these river systems (Fig. 2 and Supplementary Material 2). We use the logarithmic ratio of the sum of XRF-scanner counts per second for the typical clay mineral-bound elements aluminum (Al), potassium (K), iron (Fe), and titanium (Ti) divided by the counts per second for calcium (Ca) as a proxy for riverine terrigenous discharge (abbreviated as Log(Terr/Ca)). The selection of terrigenous elements excludes elements typically transported in aeolian dust (Zr and Si in larger quartz grains) or bound in marine biogenic opal (Si). The selection of clay-bound terrigenous elements is outlined in Supplementary Material 2 and Supplementary Figs. S6–S8. Log(Terr/Ca) tracks the relative contribution of the riverine terrigenous discharge normalized against the marine biogenic carbonate flux, which does not exhibit marked glacial–interglacial variability in this tropical region, where glacial–interglacial differences in sea surface temperatures^27,32^ and carbonate accumulation rates^25,29,31,32^ are relatively small (Supplementary Material 2 and Supplementary Figs. S9 and S10).

**Fig. 5:**
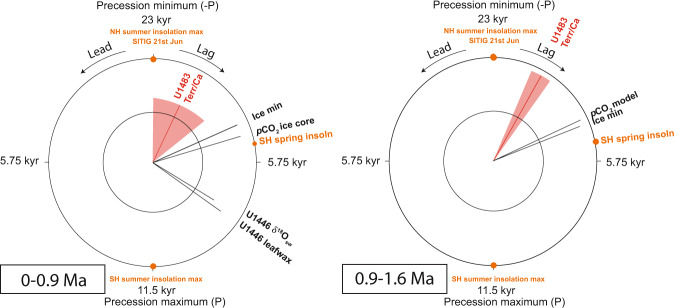
Phase wheel summaries of cross-spectral coherence and phase relationships at the precession band from 0 to 0.9 Ma and from 0.9 to 1.6 Ma. Zero phase is set to 21st June perihelion. Clockwise phases represent lags, and counterclockwise phases represent leads. Inner circle denotes coherency levels of k > 0.48 (confidence interval >90%) at 0.00556 bandwidth. The length of each vector corresponds to coherency (0 at center, 1 at circumference). Shaded areas delineate error margins for U1483 records. NH insolation = 21st June insolation at 65°N; SH insolation = 21st December insolation at 65°S; SITIG 21st June = boreal summer intertropical insolation gradient (21st June insolation at 23°N minus 21st June insolation at 23°S); ice min = δ^18^O min of LR04 stack^38^. U1446 δ^18^O_sw_ and leaf wax records are from ref. ^63^; *p*CO_2_ ice core data (0-0.8 Ma) from ref. ^118^; modeled atmospheric *p*CO_2_ from ref. ^36^.

**Fig. 6:**
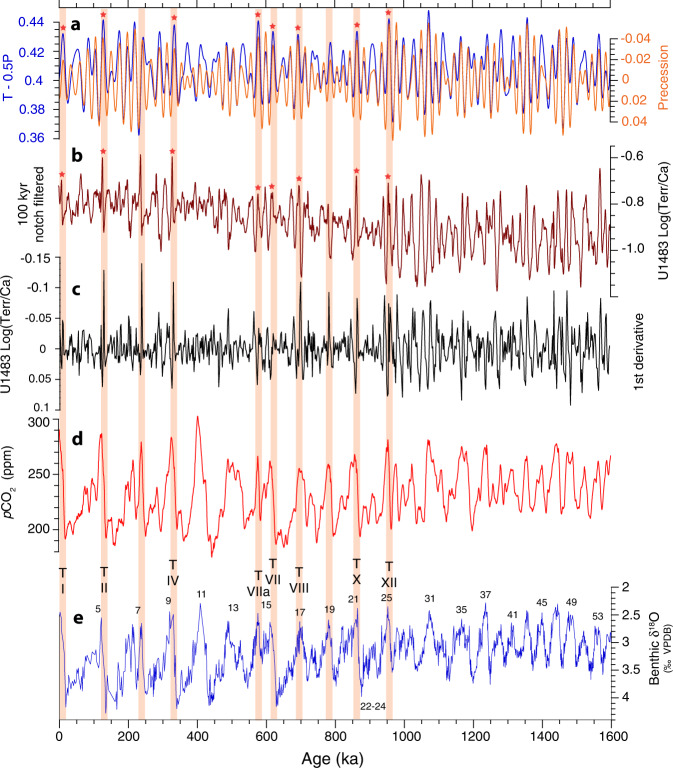
Enhanced response of Australian Summer Monsoon associated with high-latitude warming, rising atmospheric *p*CO_2_, and ice-sheet collapse during the final stage of glacial terminations following the Middle Pleistocene Transition. **a** Composite of obliquity (T) and precession (−0.5 P) and precession parameter with maximum in NH summer solstice (-P) from ref. ^119^. Red stars mark intensification of monsoonal rainfall at the end of Terminations (T) I, II, IV, VIIa, VII, VIII, X, and XII under the combined insolation forcing of obliquity maxima and precession minima. **b** U1483 Log(Terr/Ca) after removal of low-frequency variability using Gaussian notch filter with bandwidth of 0.01 centered at the frequency of 0.01 (100 kyr wavelength). **c** First derivative of the monsoonal discharge proxy Log(Terr/Ca). Units are changes of Log(Terr/Ca) per kyr, where negative numbers are standing for increase and positive number for decrease, since time is given in kyr ago (values decrease with time). **d** Modeled atmospheric *p*CO_2_ from ref. ^36^. **e** U1483 benthic foraminiferal δ^18^O with data from 0 to 410 ka from ref. ^31^; numbering refers to Marine Isotope Stages (MIS); Termination numbering follows ref. ^120^. Orange shadings mark intensification of monsoonal rainfall at the end of glacial terminations.

Log(Terr/Ca) closely follows orbital precession (-P) in the older part of the record until ~0.95 Ma, but exhibits a relatively abrupt switch to mixed 100, 41, 23, and 19 kyr variability during MIS 24 (Figs. 2 and 3). This distinct change is observed in the REDFIT and wavelet power spectra of both Log(Terr/Ca) and K (weight %), which reveal a dominant precessional (23 and 19 kyr) signal (significance level >99%) between ~1.6 and 0.95 Ma and a combined 100, 41, 23, and 19 kyr variability between ~0.95 Ma and today (Figs. 3 and 4 and Supplementary Figs. S11 and S12). The precessional signal in the Log(Terr/Ca) series is dampened during intensified glacial stages after ~0.95 Ma, but the in-phase relationship to precession (-P) remains unchanged over the past 1.6 Ma (Fig. 5 and Supplementary Fig. S13). After ~0.95 Ma, prominent increases in Log(Terr/Ca) at coeval minima in precession and maxima in obliquity correspond to ice volume decreases during the terminal phases of glacial terminations (Fig. 6).

Coherence and phase relationships of the ASM discharge record to insolation forcing and to potential internal drivers (atmospheric *p*CO_2_, ice volume) are examined in Fig. 5, Supplementary Table S2, Supplementary Figs. S13–S15. Log(Terr/Ca) exhibits high coherence and a consistent small phase lag (~2 kyr) to precession (-P, corresponding to NH summer insolation maxima). However, the amplitude variability of the Log(Terr/Ca) signal differs from that of precession (Figs. 2 and 3). Internal drivers plot in the same quadrant of the phase wheel as the ASM, but exhibit consistent phase lags of ~3 kyr to the ASM and ~5 kyr to -P (Fig. 5). The in-phase relationship to -P in our Southern Hemisphere (SH) record supports that internal feedbacks influence ASM variability, as has been previously shown for suborbital high-resolution records across the last glacial termination^29^.

### Two-step re-organization of Australian Winter Monsoon across the MPT

The productivity (RABD_660_) and bottom water oxygenation proxy (Log(Mn/S) and U) data from Site U1483 provide the first continuous, long-term record of AWM variability over the past 1.6 Myr (Fig. 2). Between 1.6 and 0.95 Ma, the RABD_660_ and Log(Mn/S) proxy records are highly coherent with orbital obliquity, whereas they are characterized by mixed 41 and 100 kyr variability after ~0.95 Ma, following the intensification and increased duration of glacial–interglacial cycles (Fig. 3 and Supplementary Figs. S4 and S5). Productivity and export flux to the seafloor increased, driving decreases in bottom water oxygenation during glacial stages through these two periods. We tentatively relate these changes to strengthening of the SH limb of the Hadley cell and the intensification of SH winter Trade winds during glacial periods, which promoted upper ocean mixing and nutrient availability.

Modeling studies suggested that the 41 kyr obliquity signal in low-latitude climate records is linked to variations in the inter-hemispheric insolation gradient inducing large-scale atmospheric circulation changes^40–42^. After ~0.95 Ma, the expansion of grounded ice sheets and sea ice with different albedo and heat exchange feedbacks during more prolonged glacial phases would have intensified latitudinal thermal gradients and strongly impacted the expansion and strength of the Hadley cell. In addition, exposure of the Sahul Shelf during intensified glacials would likely have inhibited the Holloway Current (Fig. 1), thus enhancing upper ocean mixing and productivity in the Timor Sea and, accentuating the ~100 kyr variability in the AWM proxy records. In short, the dominance of glacial–interglacial variability in the U1483 proxy records over the past 1.6 Myr underlines the sensitivity of the AWM to extratropical thermal forcing. To date, the underlying processes and pathways involved in the energy transfer between these distant regions are, however, not well-constrained, and integration into a global energy budget framework remains an outstanding challenge^7,43^.

The switch from dominant 41 kyr periodicity to mixed 41 and 100 kyr variability in the productivity and deep-water oxygenation proxy records occurred in two main steps at ~1.1 and ~0.95 Ma (Figs. 2–4 and Supplementary Figs. S4 and S5). The high-resolution AWM proxy records Log(Mn/S) and RABD_660_ display a precessional signal during the transitional period, unlike the dominant 41 kyr periodicity prior to ~1.1 Ma and the mixed 100 and 41 kyr variability after ~0.95 Ma (Fig. 2 and Supplementary Figs. S4 and S5). This transient phase was associated with asymmetric warming in the Southern Ocean and cooling in the North Pacific Ocean (Fig. 4 and Supplementary Fig. S16). In the SH, the mid-to high-latitudes temporarily warmed between ~1.1 and 0.95 Ma, as shown by elevated SSTs in the Tasman Sea^44,45^, in the subantarctic Atlantic Ocean^46,47^ and at locations close to the Antarctic margin^48^. By contrast, the latitudinal temperature gradient in the North Pacific Ocean increased and the northern boundary of the IPWP contracted, as the high-latitudes cooled (Supplementary Material 3) and the ITCZ moved southwards^49–51^. Southern Hemisphere warming during this transitional period would have reduced the SH latitudinal thermal gradient and weakened the Hadley circulation^52,53^, enhancing low-latitude feedbacks. The precessional response displayed by the AWM proxy records between 1.1 and 0.95 Ma suggests higher sensitivity to low-latitude insolation forcing during this central phase of the MPT. The two-step re-organization of the AWM occurred within a wider context of ocean–atmosphere circulation changes^54–57^, which were likely instrumental in ultimately ushering in a new mode of climate variability after ~0.95 Ma.

### Changing response of Australian Summer Monsoon to external and internal forcing after 0.95 Ma

When predicting the ASM response to future climate warming, a fundamental question remains the relative role of external (insolation) forcing versus internal feedbacks in driving monsoonal variability, especially across climate transitions associated with major changes in global temperatures, ice volume and atmospheric *p*CO_2_. Our highly resolved record of XRF-scanner-derived terrigenous discharge from Site U1483 allows insights into the changing sensitivity of the ASM to insolation forcing across the MPT. Insolation forcing controls monsoon variability through changes in the spatial distribution of insolation affecting the latitudinal thermal gradients and the transfer of heat/moisture within the tropics and between hemispheres. The role of orbital precession in driving the seasonality, spatial extent and intensity of monsoonal winds and precipitation has been extensively discussed for NH monsoonal subsystems^42,57–59^, but remains under-explored for the SH component of the Austral–Asian Monsoon.

The dominant precessional (23-19 kyr) signal in the Log(Terr/Ca) terrigenous discharge proxy record between ~1.6 and 0.95 Ma at Site U1483 (Figs. 2–3) indicates that the ASM primarily responded to external insolation forcing. Log(Terr/Ca) does not exhibit a linear relationship to precession, which can be explained by the fact that multiple factors influence the erosion, transport and deposition of sediments offshore. These factors include the amount and seasonality of monsoonal rainfall in the upstream catchment, the regional vegetation cover and the dynamics of marine tidal currents during transport across the shelf. The Log(Terr/Ca) signal is, however, highly coherent and phase-locked with precession (Fig. 5 and Supplementary Fig. S13A,S13B), demonstrating that precession is a fundamental driver of the monsoonal terrigenous input at Site U1483.

The occurrence of maximum monsoonal sediment discharge during the late stage of terminations and early part of interglacials after ~0.95 Ma (Fig. 2), when the distance between Site U1483 and the adjacent river mouths was at its greatest, clearly excludes distance to the coastline as the main control on terrigenous sediment accumulation at this site. For instance, the main discharge pulse during the last glacial termination started at 13 ka (about 1 kyr after the main meltwater pulses), when sea level was already high^29,60^. If sea level had been a main control on terrigenous accumulation at Site U1483, one would expect the highest accumulation of terrigenous sediment to occur during glacials, when the site was in a proximal location relative to the riverine source. The high volume of fine-grained clay during peak monsoonal periods and efficient transport dynamics across the shelf to the continental margin location of Site U1483 by tidal currents appear to have counteracted the sea-level influence, enabling high accumulation of terrigenous sediment at the more distal location of Site U1483.

**Fig. 7:**
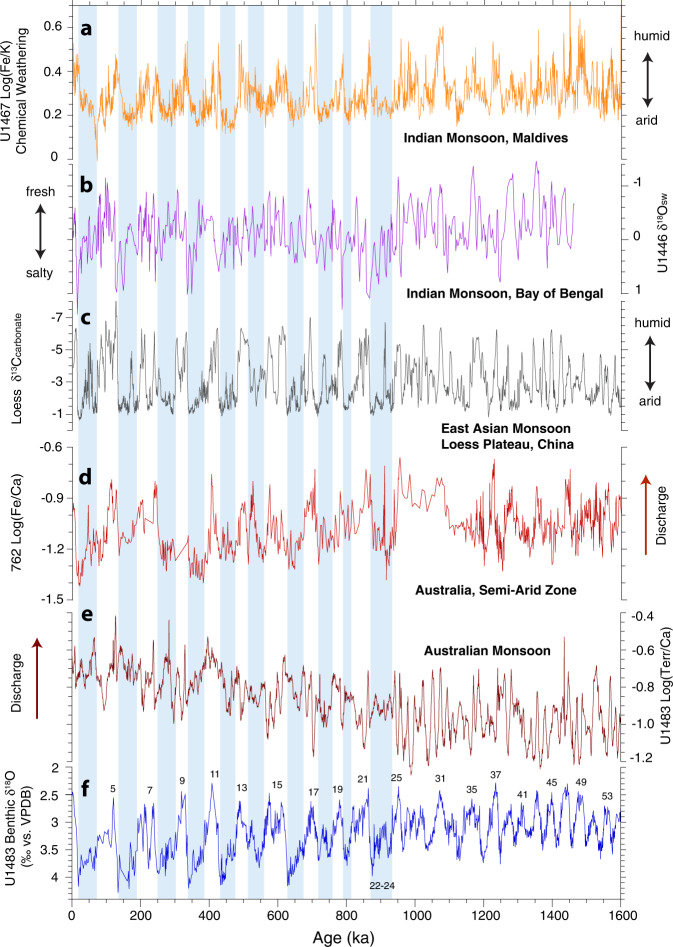
Comparison of proxy records for Indian, East Asian, and Australian Summer Monsoon over the past 1.6 Myr. **a** Chemical weathering proxy Log(Fe/K) from Site U1467^64^. **b** Salinity proxy δ^18^O_sw_ from Site U1446^63^. **c** Precipitation proxy (carbonate δ^13^C) from Chinese Loess Plateau^65^. **d** Riverine discharge proxy (Log(Fe/Ca) from Site 762^61,62^. **e** Riverine discharge proxy Log(Terr/Ca) from Site U1483. **f** Benthic foraminiferal δ^18^O from Site U1483 including data from 0 to 410 ka from ref. ^31^; numbering refers to Marine Isotope Stages (MIS). Glacial stages after 950 ka are shaded blue.

The switch to mixed 100, 41, 23, and 19 kyr variability after ~0.95 Ma (Fig. 3) reveals enhanced sensitivity of the ASM to internal drivers following the onset of high-amplitude ~100 kyr glacial–interglacial cycles. Our results add to the line of evidence that regional monsoons across the Austral–Asian Monsoon domain became increasingly forced by ice volume and *p*CO_2_-related feedbacks following the MPT, as demonstrated by the dominant ~100 kyr variance in monsoonal records from the Exmouth Plateau (eastern Indian Ocean)^61,62^, Bay of Bengal^63^, Maldives^64^, and Loess Plateau^65^ after ~0.95 Ma (Fig. 7 and Supplementary Fig. S17). The inception of longer and more intense glacial phases after ~0.95 Ma resulted in higher-amplitude changes in ice volume and atmospheric *p*CO_2_, which exerted a dominant control on inter-hemispheric temperature gradients, moisture and energy budgets and the latitudinal displacement of the ITCZ.

The switch from dominant precessional cyclicity to combined 100, 41, 23, and 19 kyr variability in the ASM terrestrial discharge was relatively abrupt and synchronous with a major decrease in the amplitude variability of precession after ~0.95 Ma (Figs. 2–4). This rapid transition was also coeval with the AWM shift from 41 kyr periodicity to mixed 41 and 100 kyr variability, indicating that a fundamental re-organization of tropical climate occurred after ~0.95 Ma. These changes also mark the onset of a prolonged interval of sustained high δ^18^O, including the failed termination at the MIS 24-23 transition^66^. Stepwise glacial expansion across MIS 24–22 culminated with an abrupt increase in ice volume at ~0.9 Ma during MIS 22, attributed to the transition from land- to marine-based ice sheets on Antarctica^67^. This pivotal phase of the MPT has been previously associated with changes in deep ocean CO_2_ storage^55,68–70^, variations in ice-sheet dynamics and atmospheric *p*CO_2_^67,71–74^ and re-organizations of the ocean–atmosphere circulation^54,56,75^. This cascade of events suggests crossing of critical thresholds during MIS 24–22, marking a turning point in long-term global climate evolution.

### Phasing of monsoonal discharge to precessional insolation forcing

The Site U1483 terrigenous discharge records exhibit a consistent response to external insolation forcing with high coherence and a ~2 kyr lag relative to -P over the past 1.6 Myr (Fig. 5). The ~2 kyr phase lag to -P is shorter than in South Asian Summer Monsoon proxy records from the Arabian Sea^6^ and the Bay of Bengal^63^, where it is in the range of ~8 kyr (Fig. 5, Supplementary Table S2, and Supplementary Fig. S13). The extended phase lag in these regions has been explained by increased heat storage in the subtropical southern Indian Ocean during enhanced SH summer insolation at high precession. This stored energy becomes available in the following months for latent heat export to the NH, implemented by the thermal contrast of cool southerly winds blowing over warm ocean waters^63^.

By contrast, the latent heat and moisture supply for the ASM, which mainly originates from the NH tropics (NE Indian Ocean, Bay of Bengal, Andaman Sea and Indonesian Seas/Maritime Continent) (Fig. 1), is primarily influenced by NH insolation. The high coherence and in-phase behavior of the terrigenous discharge proxy records to NH insolation could be explained through changes in the strength of the cross-equatorial outflow in energy and precipitation from the source area to the NW Australian region^76^. The small phase lag to -P (~2 kyr) may be partly introduced in response to internal ice volume and greenhouse gas forcings, which have a phase lag to -P of up to 5 kyr^77^, Supplementary Fig. S14, and Supplementary Table S2). This lead of the ASM over *p*CO_2_ and ice volume may be caused by the earlier onset and intensification of monsoonal rainfall, when a threshold in atmospheric *p*CO_2_ and/or ice volume/sea level is reached.

A further consideration, however, is that the Walker circulation, which determines tropical Pacific zonal temperature gradients, is an important driver of Indo-Pacific equatorial hydroclimate and ASM variability through the modulation of ENSO activity^77,78^. For instance, weaker ASM as a result of reduced heat and moisture transfer from a cooler IPWP is a common feature during El Ninõ years^30,79,80^. Proxy reconstructions and model simulations also indicated that the variability of the Walker circulation is tightly coupled to the evolution of the IPWP heat content at the precessional band^81,82^. At precession minima (-P), corresponding to warm NH summers and warm SH springs, the IPWP warms and expands, suppressing El Niño events. Maximum NH summer insolation at -P and an increased zonal upper ocean thermal contrast promote strengthening of the Walker Circulation and reduction of El Niño activity, leading to warming and increased precipitation over the entire IPWP, including the ASM region. Precessionally driven fluctuations in the IPWP heat storage and in ENSO activity, therefore, offer a plausible explanation for the robust coupling of ASM variations to NH insolation (with a phase lag of ~2 kyr to -P) over the last 1.6 Ma.

Modern observations and compilations of upper ocean heat storage data over the last glacial cycle indicated that the IPWP heat content is additionally influenced by the northwestward advection of Antarctic intermediate waters (AAIW) with a high oxygen and low nutrient content^83–87^. In analogy with the seasonal thermal development of the IPWP, precessionally driven temperature changes in advected SH thermocline waters reinforce NH induced temperature changes, amplifying the precessional signal of the IPWP heat content. At precession maxima, when austral winter insolation is weak and the SH mid-latitudes are cold, cooling from advected AAIW is transferred into the IPWP thermocline. This results in an overall cooling of the IPWP, as NH summers remain relatively cool at precession maxima. Conversely at precession minima, when SH winter insolation is at a maximum and subtropical thermocline waters warm, a net heat transport into the IPWP thermocline occurs through the shallow overturning circulation. In this context, strengthening of the Walker circulation and the suppression of El Niño events in the early Holocene have been related to increased upper ocean heat storage in the IPWP during the precessional minimum at 11.5 ka^82,88,89^. Recent compilations of upper ocean heat content records in the IPWP for the last glacial–interglacial cycles and transient climate simulations further highlighted the importance of precessional insolation as a driver of variability for the Austral–Asian Monsoon system^90,91^. These studies suggested, in particular, that precession-modulated variations in IPWP upper ocean heat content controlled the convergence of moisture and latent heat over the past 360 kyr, thus regulating the ocean-continent hydrological cycle within the Austral–Asian Monsoon domain^90^.

### Changing sensitivity to internal boundary conditions

Following the MPT, internal feedbacks involving ice volume, *p*CO_2_ and the land-sea distribution considerably altered the response of the ASM to insolation forcing, especially under more extreme glacial boundary conditions. The U1483 terrigenous input record shows a dampened response to precessional insolation forcing after ~0.95 Ma (Figs. 3 and 6) and lacustrine, vegetation and aeolian dust records from NW Australia document widespread aridity during intensified glacials^26,92,93^, supporting model predictions of increased aridification under low atmospheric *p*CO_2_ and cool conditions^94,95^. Extensive exposure of the Sunda and Sahul shelves during intense glacials after ~0.95 Ma (Fig. 1) may have also amplified the high-latitude albedo response through ocean–atmosphere interactions affecting the strength of the Walker circulation^96–98^. By contrast, sea-level change was less than −60 m during obliquity-paced glacial–interglacial cycles prior to ~0.95 Ma so that most of the NW Australian shelf remained submerged and the land-sea configuration was not substantially altered (Fig. 1).

Our records additionally show that monsoonal rainfall increased considerably and terrigenous discharge peaked in the terminal phase of glacial terminations (Fig. 6). Strikingly, the most prominent discharge events at the end of Terminations I, II, IV, VIIa, and VII, VIII, X, and XII occurred under the combined insolation forcing of obliquity maxima with precession minima (Fig. 6). This orbital configuration gives rise to the highest summer solstice insolation in the NH and to longer summers in the SH^99^. Previous work proposed that this combined insolation forcing controlled both the onset and duration of late Pleistocene terminations because it promoted high-latitude warming, atmospheric *p*CO_2_ rise and ice-sheet collapse^66,99^. These changes in boundary conditions in the final stage of terminations favored the expansion of the tropical belt and intensification of convective monsoonal rainfall over tropical regions including NW Australia. This enhanced response during terminations contrasts markedly with the dominant precessional rhythm in the ASM proxy records prior to the MPT, demonstrating enhanced sensitivity of the ASM to high-latitude climate feedbacks following the onset of high-amplitude glacial–interglacial cycles.

Our high-resolution records of terrigenous river discharge, productivity and bottom water oxygenation from Site U1483 provide insights into the forcing processes and the evolution of the ASM and AWM across the MPT. The ASM primarily responded to precessional insolation forcing until ~0.95 Ma, but became increasingly driven by internal feedbacks following the inception of high-amplitude glacial–interglacial cycles. Over the past 1.6 Myr, Australian monsoonal discharge remained coherent with precession with a phase lag of ~2 kyr relative to –p. This small lag suggests that variability at the precessional band was closely tied to changes in IPWP temperatures and the strength of the Walker circulation. By contrast, winter monsoon proxy records primarily tracked glacial–interglacial variability over the past 1.6 Myr, exhibiting a two-step shift from dominant 41 kyr to mixed 41 and 100 kyr variability at ~1.1 and 0.95 Ma. Our results underline the changing sensitivity of the Australian Monsoon to external and internal forcings under different global ice volume and greenhouse gas boundary conditions. These findings raise the possibility that future *p*CO_2_ increase and global warming will lead to substantial strengthening of the ASM and weakening of the SH Trade winds.

## Methods

### Composite sediment splice

This study is based on a composite sediment splice from three holes drilled with the advanced piston corer (APC) system at IODP Site U1483 (13°5.24’S, 121°48.25’E; 1733 m water depth) located on the Scott Plateau off NW Australia^37^ (Fig. 1). The extended succession recovered at Site U1483 consists of carbonate- and clay-rich hemipelagic sediments, exhibiting cyclic variations on decimeter to meter scales. The lithogenic component is mainly derived from NW Australian rivers including the Fitzroy and Ord Rivers^29,100^. Detailed lithological descriptions are provided in ref. ^37^. The original shipboard sediment splice was revised, based on the X-ray fluorescence (XRF) core scanning records^101^, providing a continuous sedimentary record for the last ~1.6 Ma.

### Sampling strategy

Sediment samples (2-cm thick half-round samples) were selected in ~10 cm spacing between 363-U1483-A-5H-2W 110–112 cm and 363-U1483-A-15H-7W 50–52 cm (41.83 to 151.97 revised meter composite depth (r-mcd)) and in ~20 cm spacing between 363-U1483-B-16H-4W 60–62 cm and 363-U1483-B-16H-6W 20–22 cm (152.01 to 154.55 r-mcd) along the composite sediment splice^101^ from Holes U1483A, U1493B, and U1483C. Samples were dried at 40 °C, weighed and washed over a 63-μm sieve. The residues were dried at 40 °C on filter paper then weighed and separated in three size fractions <150, 150–250, and >250 µm.

### Stable oxygen isotopes and chronology

We selected three to eight well-preserved specimens of the benthic foraminiferal species *Cibicidoides wuellerstorfi* and/or *Cibicidoides mundulus* from the size fraction >250 μm. In 29 samples, only 1–2 specimens were analyzed, and in 12 samples the species *Uvigerina* spp. or *Hoeglundina elegans* were measured, when *C. wuellerstorfi* and *C. mundulus* were rare or absent. Tests were broken into large fragments and cleaned in ethanol in an ultrasonic bath, then dried at 40 °C. Measurements were performed with a Finnigan MAT 253 mass spectrometer coupled online to a Carbo-Kiel IV device for automated CO_2_ preparation from carbonate samples at the Leibniz Laboratory for Radiometric Dating and Stable Isotope Research, Christian-Albrechts-University Kiel. Samples were reacted by individual acid addition (99% H_3_PO_4_ at 75 °C). The external standard error is better than ±0.08‰ for δ^18^O, based on international standards. Results were calibrated using the National Institute of Standard and Technology (NIST) (Gaithersburg, Maryland) carbonate isotope standard NBS (National Bureau of Standard) 19, and the international carbonate standard IAEA (International Atomic Energy Agency) 603 and reported on the Vienna PeeDee Belemnite (VPDB) scale. Duplicate measurements of 32 samples show an average standard deviation of 0.08‰ for δ^18^O. We applied a correction factor of −0.64‰ to *Uvigerina* δ^18^O (following refs. ^102,103^) and of −1.26‰ for *Hoeglundina* δ^18^O (following ref. ^104^) to normalize values to *C. wuellerstorfi* δ^18^O. The Site U1483 age model is based on correlation of the benthic foraminiferal δ^18^O to the LR04 oxygen isotope stack^38^, using 62 tie points (Supplementary Table S1 and Supplementary Fig. S1). Tuning of the U1483 record to the LR04 stack was performed with AnalySeries 2.08^105^.

### X-ray fluorescence core scanning

We performed high-resolution X-ray fluorescence (XRF) core scanning with the 2^nd^ Generation Avaatech XRF Core Scanner at the Institute of Geosciences, Christian-Albrechts-University, Kiel (Germany). The archive halves were equilibrated to room temperature before scanning and a thin layer of sediment was removed from the top to obtain a fresh, even surface for scanning. We scanned at 2 cm intervals along the shipboard splice with approximately 1–2 m overlaps at splice tie points. Scanning was performed with 10 kV (750 µA, 10 s acquisition time, no filter) and 30 kV (2000 µA, 20 s acquisition time, Pd-thick filter) on the archive halves, which were covered with a 4 µm thick Chemplex Prolene Thin-Film foil to prevent contamination of the XRF detector. We used a crosscore slit size of 1.2 cm and a downcore slit size of 1 cm. The data reported here were acquired by a XR-100CR detector from Amptek and an Oxford Instruments 50 W XTF5011 X-Ray tube with rhodium (Rh) target material. Raw X-ray spectra were converted into area counts using the iterative least-square software package WIN_AXIL from Canberra Eurisys and a core-specific model. The elements Al, Si, S, K, Ca, Ti, Mn, and Fe were analyzed with the 10-kV setting, and the elements Br, Rb, and Zr with the 30-kV setting. The measured area counts per second of the spectral peaks of each element were transferred to logarithmic elemental ratios, which provide the most easily interpretable signals of relative changes in chemical composition. The use of elemental ratios minimizes the risk of measurement artifacts from variable signal intensities due to changes in sediment density, pore volume, water content, and matrix effects^106^.

### Reflectance spectroscopy scanning

Reflectance spectroscopy of the archive section halves of sediment cores was measured during IODP Expedition 363 using an Ocean Optics USB4000 spectrophotometer on the Section Half Multisensory Logger^37^. Measurements were taken at 2.5 cm spacing and reflectance values for visible wavelengths were recorded in 2 nm wide spectral bins between 380 and 900 nm. We use these in-situ visible light reflectance spectroscopy data to analyze the abundance of diagenetic photosynthesis pigments (chlorins) in bulk sediments along the composite sediment splice from Holes U1483A, U1483B and U1483C^101^, expressed in the relative absorption at the 660 nm spectral band. Chlorin concentration is highly correlated to marine organic carbon content, organic matter preservation, and marine primary productivity^107,108^. The relative absorption band depth at 660 nm was calculated using the algorithm from ref. ^108^:

$${{{{{{\rm{RABD}}}}}}}_{660}=\left\{\left[\left(6*{{{{{{\rm{R}}}}}}}_{590}+7*{{{{{{\rm{R}}}}}}}_{730}\right)/13\right]/{{{{{{\rm{R}}}}}}}_{660}\right\}/{{{{{{\rm{R}}}}}}}_{{{{{{\rm{mean}}}}}}}$$

where RABD_660_ is the relative absorption band depth at 660 nm, R_590_, R_660_, and R_730_ the reflectance at 590, 660, and 730 nm wavelength and R_mean_ the mean reflectance over the interval 590–730 nm. Concentrations of chlorophyll are preserved in sediments as chlorins, the pigment-transformation products of chlorophyll. The efficacy of chlorin concentrations as a proxy for total primary productivity variations in Pleistocene records was initially demonstrated for Pleistocene sediments from the subtropical Atlantic continental margin^107^, and a quantitative link between chlorophyll and chlorin concentrations in seafloor sediments was recently established on a global scale^109^. These results show that chlorins can be used as quantitative proxies of total primary productivity in paleo-reconstructions for regions of high and moderate primary productions, where sea surface chlorophyll alpha concentrations are above 0.2 µm/l, which is the case for the Timor Sea region^109–111^.

### Spectral natural gamma ray

Spectral gamma ray was measured with the Natural Gamma Ray spectrometer on R/V JOIDES Resolution during IODP Expedition 363^37^. The system consists of eight detectors that measure the intensity of gamma radiation at 1024 different energy levels, between 0 and 3000 keV simultaneously at adjacent intervals of the core to minimize counting times and statistical error due to the limited measurement time available during drilling expeditions^112^. The system acquires spectra with sufficient energy to be post-processed for elemental abundances of ^40^K, ^238^U, and ^232^Th. Performance of the instrumental setup and processing algorithm were evaluated by comparing results to shore-based inductively coupled plasma-mass spectrometry (ICP-MS), inductively coupled plasma-emission spectrometry (ICP-ES), and quantitative wavelength-dispersive X-ray fluorescence (XRF) analyses with linear regression R^2^ values between 0.89 for K and Th and 0.84 for U^39^.

### Time series analysis

Spectral analysis was performed on the unevenly spaced time series of the U1483 benthic foraminifera δ^18^O, XRF-derived elemental log ratios, reflectance spectroscopy RABD_660_ and natural gamma ray data between 400 and 1600 ka and between 0 and 1600 ka by combining our data with the records of ref. ^31^. We used the REDFIT function, an implementation of the REDFIT procedure of ref. ^113^ with a Blackman–Harris window of two segments and a frequency oversampling value of 2 in the PAST 4.10 software^114^. Significance lines of 99%, 95%, 90% confidence intervals (CIs) are based on parametric approximation (chi-square test).

Wavelet spectral analysis was performed on evenly spaced time series between 400 and 1600 ka and between 0 and 1600 ka by combining our data with the records of ref. ^31^. Benthic oxygen data were first evenly sampled to 1 kyr resolution, and XRF-scanner-derived data and RABD_660_ were evenly sampled to 0.1 kyr resolution by linear integration using the resampling function in AnalySeries 2.08. We used the wavelet transform algorithm for evenly spaced time series analysis in 1 kyr and 0.1 kyr temporal resolution with a Morlet basis function in the PAST 4.10 software^114^. The *P* = 0.05 significance level derived by a chi‐square test is displayed as a black contour line and a cone of influence indicates the area with possible boundary effects^115^.

## Supplementary information

Supplementary Information

## Data Availability

The datasets generated and analyzed in this study are available at the Data Publisher for Earth & Environmental Science PANGAEA (10.1594/PANGAEA.948036).
