# Supplementary material for: Middle Pleistocene re-organization of Australian Monsoon
Source: Nat Commun. 2023 Apr 10;14:2002. doi: 10.1038/s41467-023-37639-x (PMC10086051; doi:10.1038/s41467-023-37639-x)
Supplement: Supplementary file 1 — Supplementary Information [file 41467_2023_37639_MOESM1_ESM.pdf]

Middle Pleistocene re-organization of Australian Monsoon

Li Gong[1], Ann Holbourn[1], Wolfgang Kuhnt[1], Bradley Opdyke[2], Yan Zhang[3], Ana Christina Ravelo[3], Peng Zhang[4], Jian Xu[4], Kenji Matsuzaki[5-6], Ivano Aiello[7], Sebastian Beil[1], Nils Andersen[8]

[1]*Institute of Geosciences, Christian-Albrechts-University, D-24118 Kiel, Germany.*

[2]*Research School of Earth Sciences, Australian National University, Mills Road, Acton, ACT 2601, Australia.*

[3]*Ocean Sciences Department, University of California, 1156 High Street, Santa Cruz, CA 95064, USA.*

[4]*Institute of Cenozoic Geology and Environment, State Key Laboratory of Continental Dynamics and Department of Geology, Northwest University, Xi'an 710069, China.*

[5]*Atmosphere and Ocean Research Institute, The University of Tokyo, 5-1-5, Kashiwanoha, Kashiwa, Chiba 277-8564, Japan.*

[6]*Department of Earth and Planetary Science, Graduate School of Science, The University of Tokyo, 7-3-1, Hongo, Bunkyo-ku, Tokyo 113-0033, Japan.*

[7]*Department of Geological Oceanography, Moss Landing Marine Laboratories, San Jose State University, Moss Landing, CA 95039, USA.*

[8]*Leibniz Laboratory for Radiometric Dating and Stable Isotope Research, Christian-Albrechts-University Kiel, D-24118, Kiel, Germany.*

Corresponding author: Ann Holbourn; email: ann.holbourn@ifg.uni-kiel.de

SUPPLEMENTARY MATERIAL 1-4

Supplementary Material 1: Age Model

The age model between 41.83 and 154.55 revised meter composite depth[1] (r-mcd) is based on correlation of the Site U1483 benthic foraminiferal $\delta^{18}O$ to the LR04 oxygen isotope stack[2] (Supplementary Figure S1). Between the core top and our uppermost tie point at 42.03 r-mcd (424 ka), we used the age model in ref. 3. Details of the 62 tie points are provided in Supplementary Table S1.

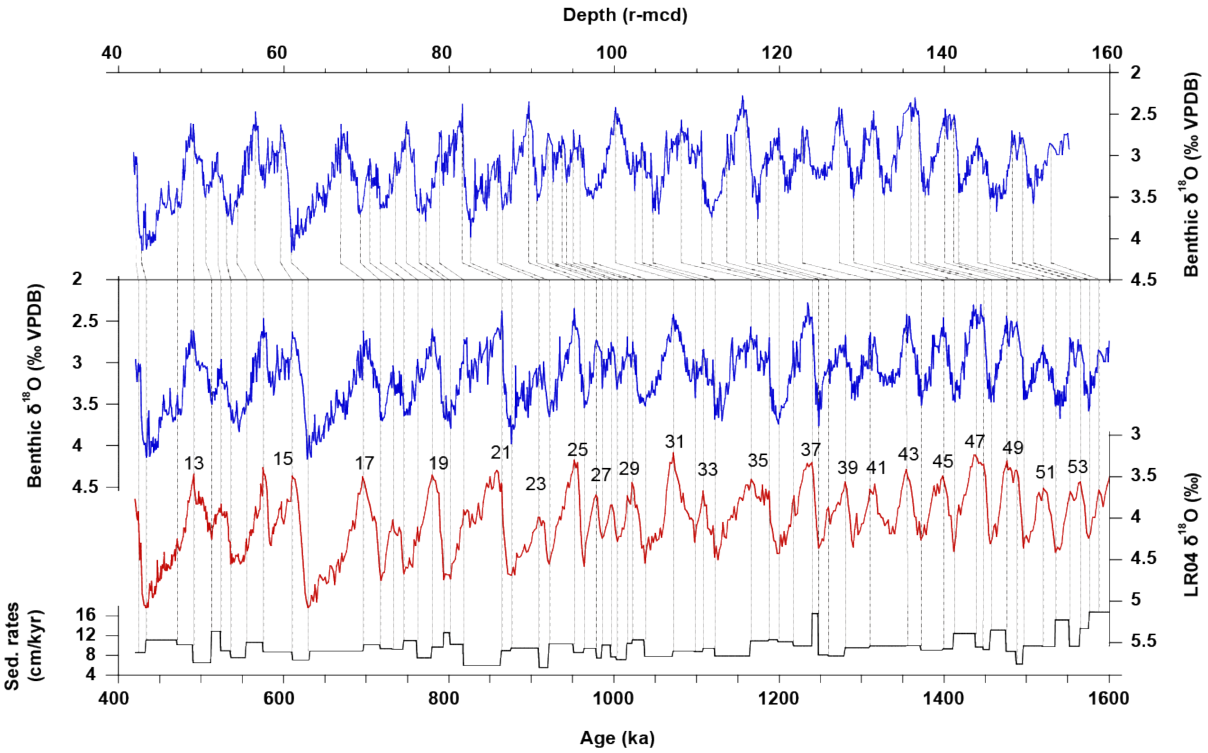

Supplementary Figure S1. Correlation of U1483 benthic foraminiferal $\delta^{18}O$ (blue) to LR04 $\delta^{18}O$ stack[2] (red). Dashed black lines indicate tie points used to derive age model. Numbers refer to Marine Isotope Stages. Depth unit r-mcd = revised meter composite depth from ref. 1.

Supplementary Table S1. Age tie points used for correlation of benthic foraminiferal $\delta^{18}O$ from IODP Site U1483 to LR04 $\delta^{18}O$ stack[2].

Depth (r-mcd)	Age (ka)	Tie point description
42.03	424	Oldest $\delta^{18}O$ minimum of MIS 11
42.84	433	$\delta^{18}O$ maximum of MIS 12a
47.06	471	Transient $\delta^{18}O$ minimum at the end of MIS 13
49.07	491	$\delta^{18}O$ minimum of MIS 13a
50.50	513	$\delta^{18}O$ maximum of MIS 13b
51.95	524	$\delta^{18}O$ minimum of MIS 13c
53.04	536	$\delta^{18}O$ maximum of MIS 14a
54.47	555	$\delta^{18}O$ maximum of MIS 14c
56.60	575	$\delta^{18}O$ minimum of MIS 15a
59.65	610	$\delta^{18}O$ minimum of MIS 15e
61.11	631	$\delta^{18}O$ maximum of MIS 16a
66.93	696	$\delta^{18}O$ minimum of MIS 17c
69.11	718	$\delta^{18}O$ maximum of MIS 18a
70.45	732	$\delta^{18}O$ minimum of MIS 18b
71.73	746	$\delta^{18}O$ maximum of MIS 18e
73.52	762	$\delta^{18}O$ minimum of MIS 19a
74.86	780	$\delta^{18}O$ minimum of MIS 19c
76.24	794	$\delta^{18}O$ maximum of MIS 20a
77.21	802	$\delta^{18}O$ maximum of MIS 20c
78.89	818	$\delta^{18}O$ minimum of MIS 21a
81.61	864	$\delta^{18}O$ minimum of MIS 21g
82.67	876	$\delta^{18}O$ maximum of MIS 22
85.88	910	$\delta^{18}O$ minimum of MIS 23c
86.54	922	$\delta^{18}O$ maximum of MIS 24
89.67	952	$\delta^{18}O$ minimum of MIS 25e
90.73	964	$\delta^{18}O$ maximum of MIS 26
92.05	978	$\delta^{18}O$ minimum of MIS 27
92.62	986	$\delta^{18}O$ maximum of MIS 28a
93.74	997	$\delta^{18}O$ minimum of MIS 28b
94.27	1004	$\delta^{18}O$ maximum of MIS 28c
95.13	1016	Youngest $\delta^{18}O$ minimum of MIS 29
95.83	1023	Central $\delta^{18}O$ minimum of MIS 29
97.53	1038	$\delta^{18}O$ maximum of MIS 30
100.20	1072	$\delta^{18}O$ minimum of MIS 31
102.52	1098	$\delta^{18}O$ maximum of MIS 32
103.39	1108	$\delta^{18}O$ minimum of MIS 33
104.72	1122	$\delta^{18}O$ maximum of MIS 34
108.18	1166	Central $\delta^{18}O$ minimum of MIS 35
110.60	1188	Oldest $\delta^{18}O$ minimum of MIS 35
111.82	1199	$\delta^{18}O$ maximum of MIS 36
113.78	1217	Transient $\delta^{18}O$ minimum at the end of MIS 37
116.06	1240	Oldest $\delta^{18}O$ minimum of MIS 37
117.43	1248	$\delta^{18}O$ maximum of MIS 38
118.39	1260	Transient $\delta^{18}O$ minimum at the end of MIS 39
119.97	1280	$\delta^{18}O$ minimum of MIS 39
122.83	1310	$\delta^{18}O$ minimum of MIS 41
127.23	1354	$\delta^{18}O$ minimum of MIS 43
129.05	1372	$\delta^{18}O$ maximum of MIS 44
131.44	1399	$\delta^{18}O$ minimum of MIS 45
132.71	1412	$\delta^{18}O$ maximum of MIS 46
135.92	1438	$\delta^{18}O$ minimum of MIS 47
136.86	1448	Oldest $\delta^{18}O$ minimum of MIS 47
137.69	1457	$\delta^{18}O$ maximum of MIS 48
140.13	1475	$\delta^{18}O$ minimum of MIS 49
141.23	1488	Oldest $\delta^{18}O$ minimum of MIS 49
141.75	1496	$\delta^{18}O$ maximum of MIS 50
144.11	1520	$\delta^{18}O$ minimum of MIS 51
145.60	1535	$\delta^{18}O$ maximum of MIS 52
148.20	1552	Youngest $\delta^{18}O$ minimum of MIS 53
149.46	1565	Oldest $\delta^{18}O$ minimum of MIS 53
150.87	1576	$\delta^{18}O$ maximum of MIS 54
152.83	1587	Transient $\delta^{18}O$ minimum at the end of MIS 55

Supplementary Material 2: Spectral natural gamma ray, reflectance spectroscopy and XRF scanner-derived elemental proxy data

Primary productivity and bottom water oxygenation

We use visible light relative absorbance band depth at 660 nm ($RABD_{660}$ in ~250 yr resolution) derived from color reflectance spectroscopy, XRF-scanner derived logarithmic ratio of manganese and sulfur (Log(Mn/S) in ~200 yr resolution) and spectral gamma ray derived uranium (U) concentrations (~1 kyr resolution) to monitor variations in primary productivity and in bottom water oxygenation at Site U1483.

The relative absorbance band depth at 660 nm ($RABD_{660}$) from color reflectance spectroscopy is directly related to the concentration of chlorins in the sediment, which are a decay product of chlorophyll alpha from marine primary producers (Material and Methods). Detailed studies of Total Organic Carbon (TOC) and chlorin concentrations measured in discrete samples from piston core MD01-2378, located 0.8 nmi northwest of Site U1483, revealed a marked glacial-interglacial variability over the last five glacial cycles[4-5]. The glacial-interglacial variability in chlorin concentrations closely matches that of the $RABD_{660}$ record extracted from the shipboard spectrophotometry measurements in the same core (Supplementary Figure S2A-B). Furthermore, chlorin concentrations in core MD01-2378 show a high correlation to TOC values (Supplementary Figure S2C), indicating that most of the preserved organic matter at this site is composed of chlorophyll alpha derivatives and other organic compounds of marine origin[4-5].

Bromine counts per second (Br_area cps) from XRF scanning have previously been used to estimate the marine organic carbon content in sediment cores from the Arabian and Mediterranean Seas[6] and solid phase Br has been used as a direct estimate of the marine organic matter content[7]. The U1483 Br area counts closely match variations in chlorin abundance ($RABD_{660}$) on an orbital timescale (Supplementary Figure S3), indicating that both proxies are linked to marine organic matter accumulation.

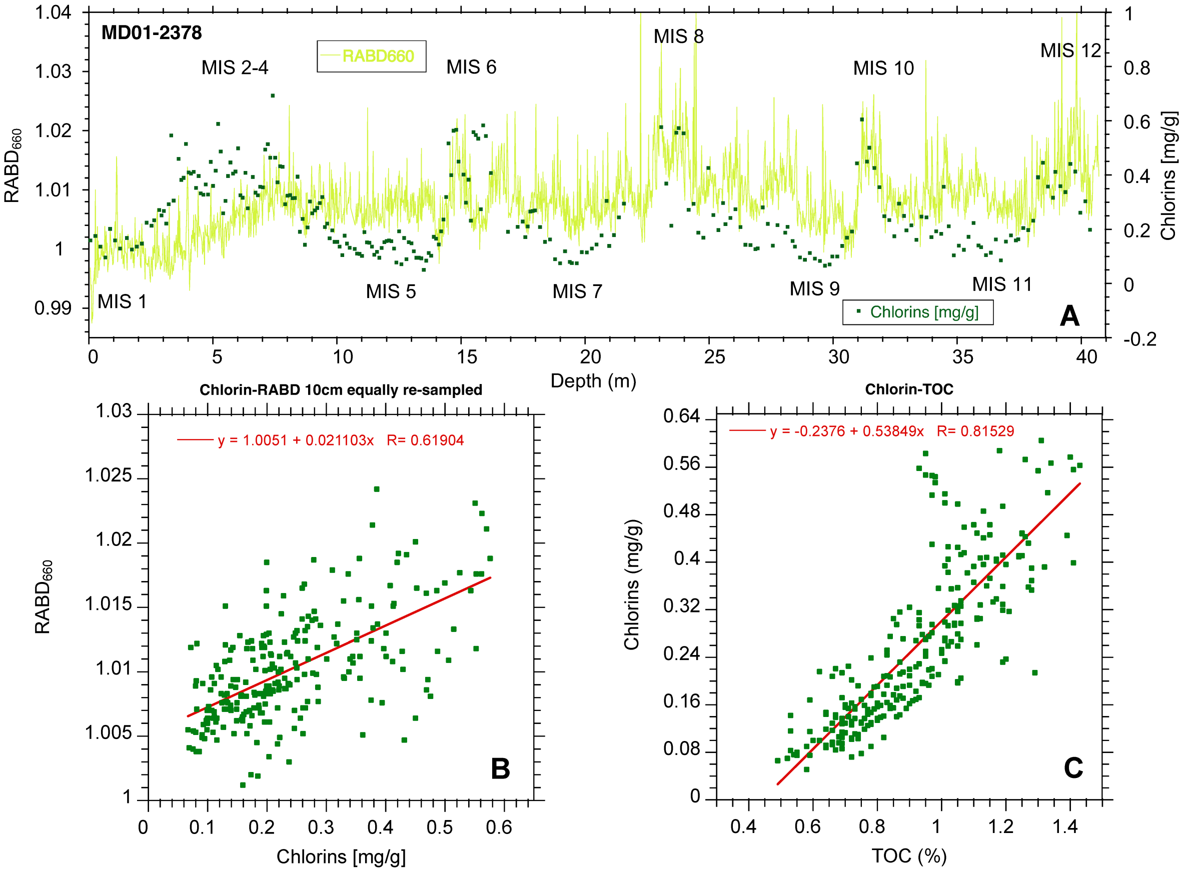

Supplementary Figure S2. Comparison of chlorin concentrations in discrete samples from core MD01-2378 and the high-resolution $RABD_{660}$ record extracted from shipboard spectrophotometry measurements in the same core over the last five glacial cycles[4-5]. **A-B**. High correlation of chlorin concentrations to total organic carbon (TOC) values. **C**. indicates primarily marine origin of the TOC. Note that regression plots (**B** and **C**) are only based on data below 7 m in core MD01-2378, to avoid known issues of sediment stretching and water content changes caused by the Calypso coring system in the upper 7 meters of the core[8]. MIS: Marine Isotope Stage.

Numerous studies have highlighted the association of elevated U concentrations with organic-matter-enriched marine sediments[9]. Uranium concentrations are frequently used as proxy indicators of organic matter preservation in marine sediments and U enrichment has been suggested as a tool to characterize suboxic marine environments[10-11]. On a global scale, more than three quarters of the total dissolved U riverine flux to the ocean is deposited in suboxic continental margin sediments that constitute the largest global U sinks[12]. In the modern ocean, U diffuses under oxygen depleted conditions from sea water into reducing sediments and remains immobile there. A recent study of surface sediments in the Barents Sea and Black Sea demonstrated that the change in redox conditions, from oxidizing to reducing, leads to an increased U content, implying that increased authigenic U concentrations provide a reliable indicator of reducing conditions at the sea floor[13]. However, the preservation of authigenic U is additionally determined by the depth of the redox zone and the sedimentation rate. In Atlantic cores with sedimentation rates <2 cm/kyr and a

deepening redox horizon, U may be re-mobilized in association with organic matter burn-down in the uppermost part of the sediment column[14]. This secondary removal of U, which mainly occurs during glacial terminations in the Atlantic, is unlikely at Site U1483, where sedimentation rates are ~10 cm. In addition, the relatively high sedimentation rates at Site U1483 decrease the potential effects of bioturbation on U concentrations.

XRF-scanner derived logarithmic ratios of the redox-sensitive elements manganese (Mn) and sulfur (S) as well as Mn and iron (Fe) exhibit similar glacial-interglacial variability as chlorin ($RABD_{660}$), Br and U concentrations (Supplementary Figures S3-S4). Authigenic Mn-oxides frequently occur in deep sea sediments, when bottom and pore waters are oxic and organic matter respiration rates are low. Under reducing conditions, Mn oxides do not precipitate and Mn concentrations in carbonates and silicates of the detritic fraction are low. With increasing oxygenation, bulk Mn concentrations increase relative to the Mn content of the detritic fraction. Normalization to typical detritic elements such as Fe or to elements that are insoluble under low oxygen conditions such as S (mainly occurring in pyrite) allows to monitor changes in the oxygenation of bottom/pore waters. The redox control on Mn accumulation is also highly dependent on its speciation. While Mn-oxides and Mn-oxyhydroxides are relatively insoluble and abundant in oxygenated environments, Mn carbonates dominate only in severely oxygen depleted environments[15]. At Site U1483, where oxic pore water conditions prevail, Mn occurs in the form of Mn-oxide coatings. We used the ratio of Mn to S as a bottom/pore water oxygenation index, since S is enriched as sulfate in barite and in reduced form as authigenic pyrite in sediments with oxygen depleted pore waters.

A distinctive feature of the U1483 Log(Mn/S) is the long-term increasing trend in the upper part of the sediment succession (Supplementary Figures S3, S5), which we relate to the influence of diagenetic processes such as compaction and related changes in pore water content. Previous studies have shown that results of XRF scanning on core surfaces deviate from quantitative discrete XRF measurements through attenuation of scanning intensities by the water content in the upper ~100 m of the marine sediment core, resulting in a gradual downcore increase in the scanning intensities of lighter elements, such as Al, Si, and S[16-17]. At Site U1483, the downcore decrease in porosity and water content markedly affects the upper ~60 m (0 to ~600 ka) and is clearly expressed in the shipboard GRAPE density data[18] and the XRF scanner chlorine (Cl) record (not shown), which is related to the salt content in the pore water.

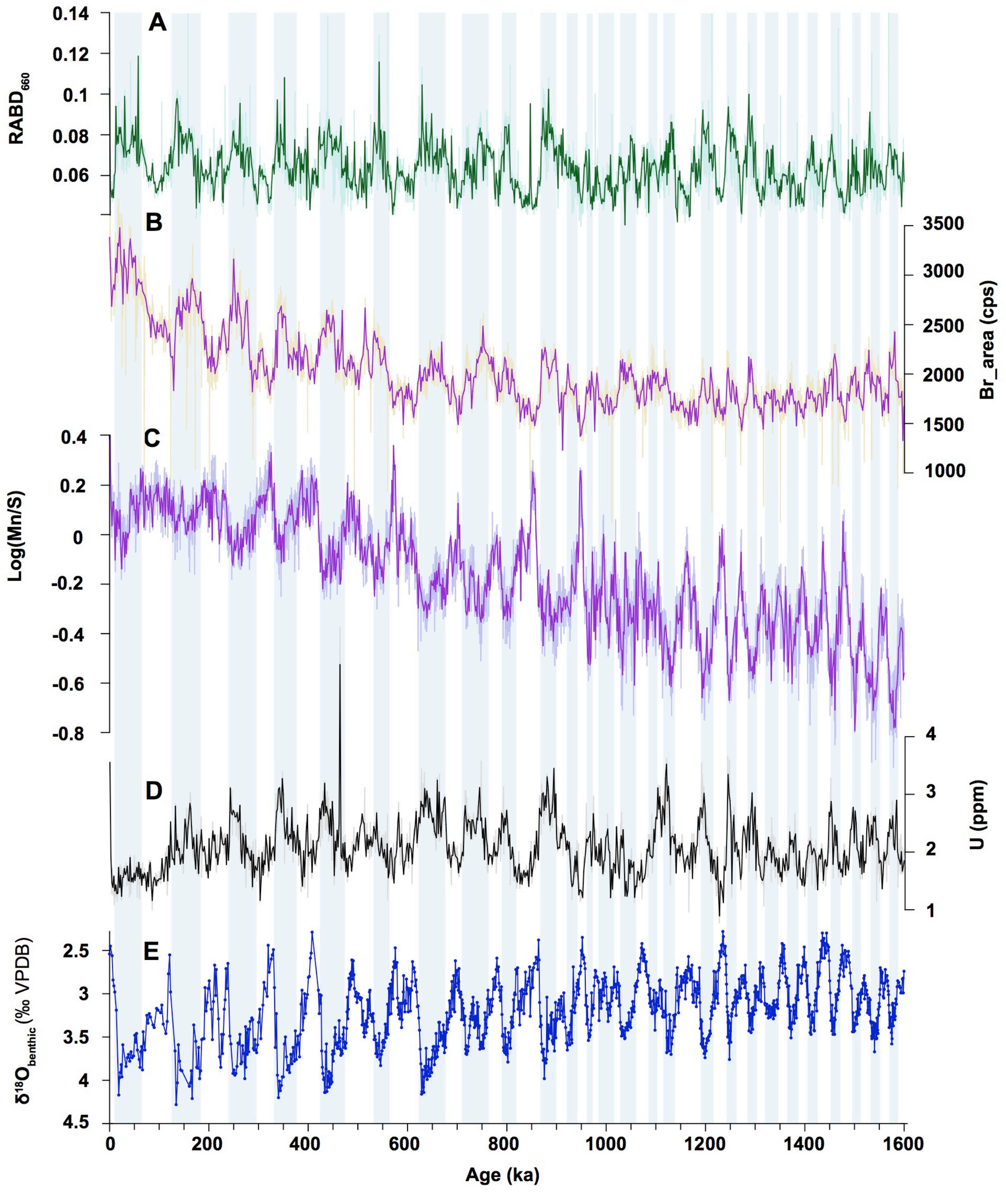

Supplementary Figure S3. U1483 proxy records of marine primary productivity and bottom water oxygenation, derived from reflectance spectroscopy, XRF-scanner and spectral natural gamma ray data. **A.** $RABD_{660}$, relative absorption band depth at 660 nm (proxy for chlorin concentration) from shipboard reflectance spectroscopy[13]. **B.** Bromine area counts per second (cps) from XRF-scanning. **C.** Logarithmic ratio of manganese and sulfur (Log(Mn/S)) from XRF-scanning. **D.** Uranium concentration estimates (ppm) from shipboard spectral gamma ray data[18-19]. **E.** U1483 benthic foraminiferal $\delta^{18}O$ with data from 0 to 410 ka from ref. 3. Thin lines show raw counts, thick lines show Stineman interpolations (interpolate function in Kaleidagraph 5.0 software). Light blue shadings indicate glacial stages. Note long-term increasing trends in Br and Log(Mn/S) after ~1 Ma related to compaction and associated changes in pore water content in the upper ~100 m of the sediment succession.

A. RABD$_{660}$

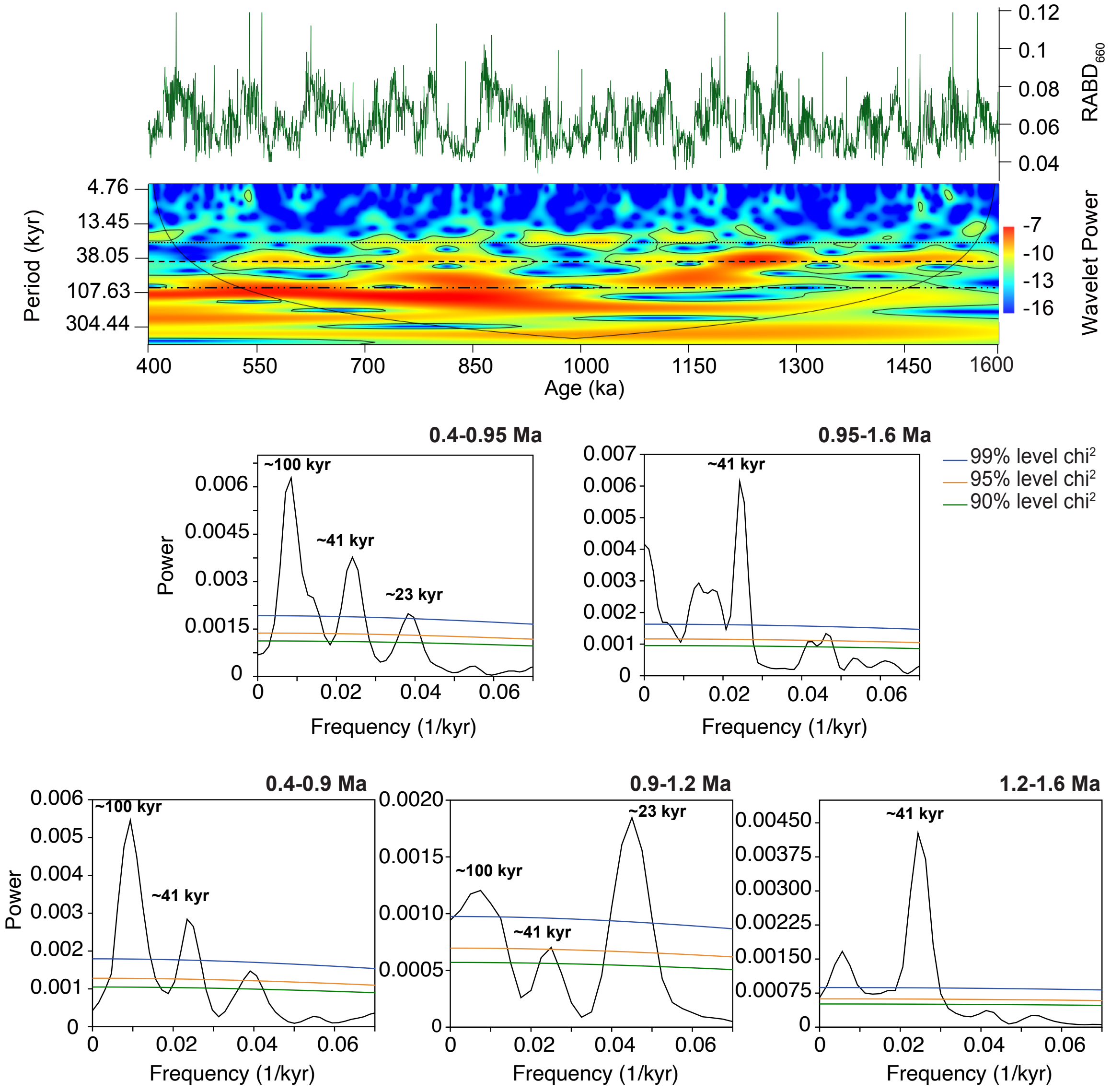

RABD660
Wavelet Power
Period (kyr)
Age (ka)
0.4-0.95 Ma
0.95-1.6 Ma
99% level chi2
95% level chi2
90% level chi2
~100 kyr
~41 kyr
~23 kyr
Power
Frequency (1/kyr)
0.4-0.9 Ma
0.9-1.2 Ma
1.2-1.6 Ma

B. Log (Mn/S)

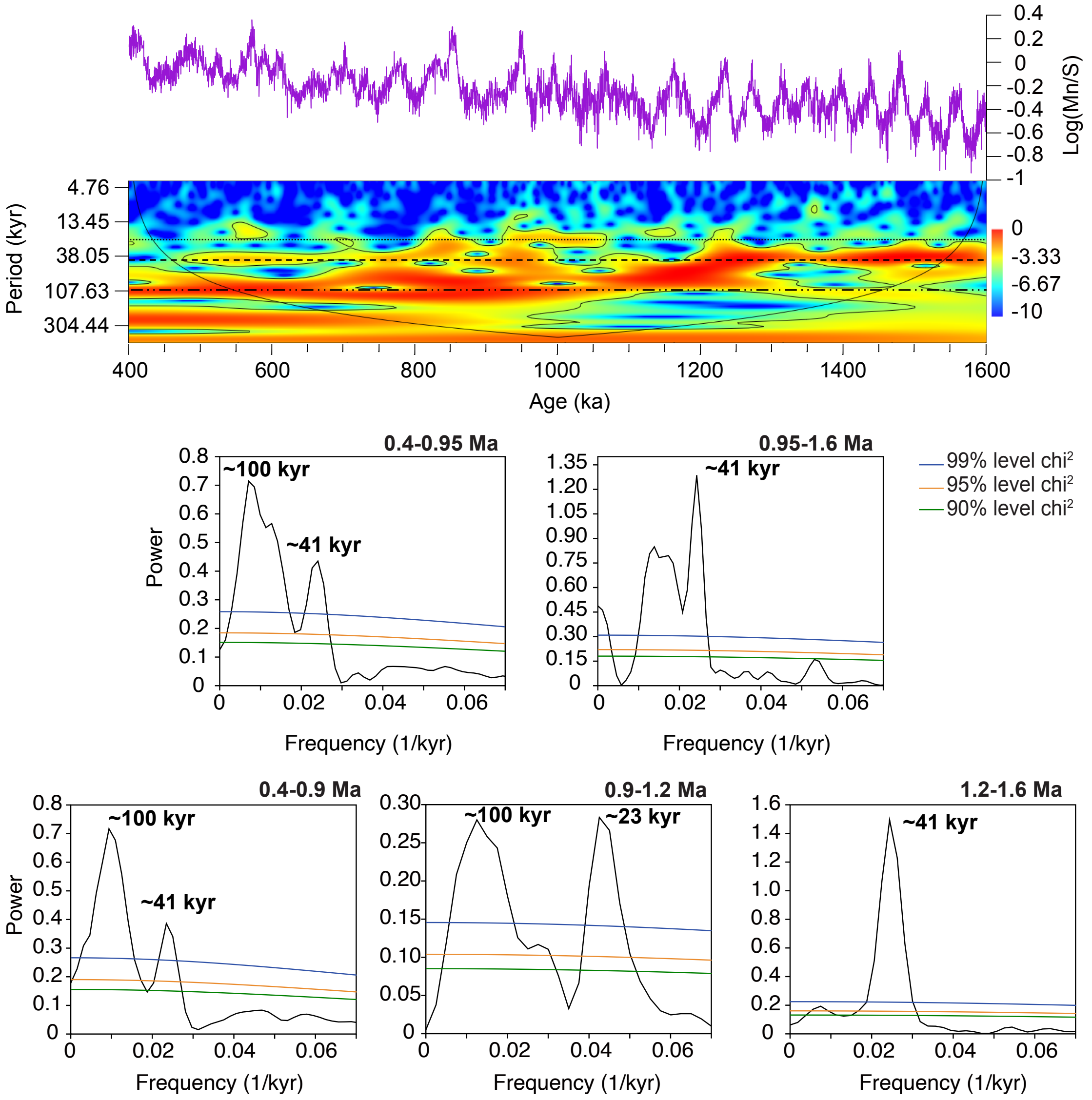

Log(Mn/S)
Period (kyr)
Age (ka)
0.4-0.95 Ma
~100 kyr
~41 kyr
0.95-1.6 Ma
~41 kyr
99% level chi²
95% level chi²
90% level chi²
Power
Frequency (1/kyr)
0.4-0.9 Ma
~100 kyr
~41 kyr
0.9-1.2 Ma
~100 kyr
~23 kyr
1.2-1.6 Ma
~41 kyr

C. U (ppm)

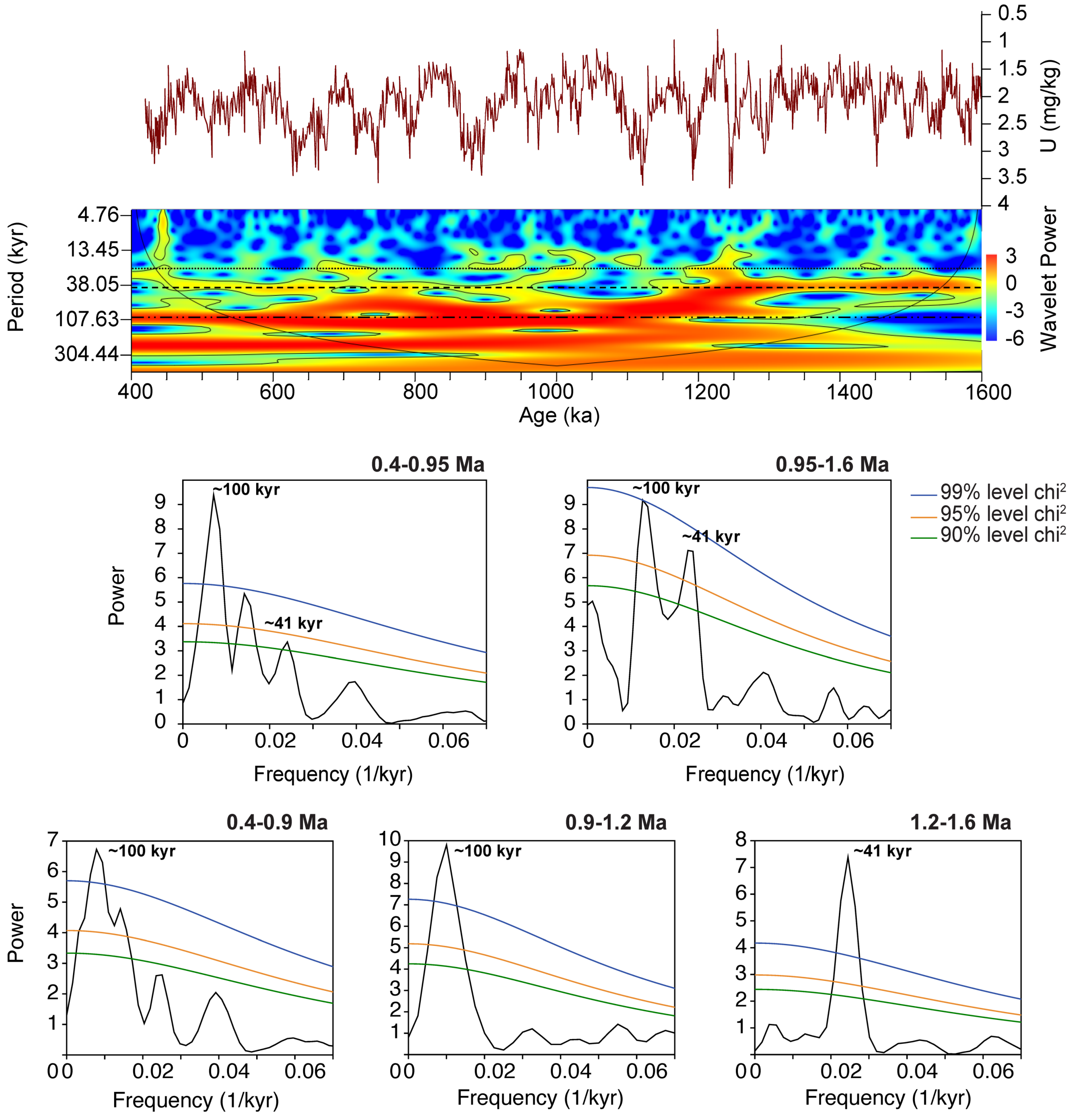

Supplementary Figure S4. Wavelet and spectral analyses of U1483 marine primary productivity and bottom water oxygenation for the time interval 1.6 to 0.4 Ma. **A.** $RABD_{660}$, relative absorption band depth at 660 nm (proxy for chlorin concentration) from shipboard reflectance spectroscopy[18]. **B.** Logarithmic ratio of manganese and sulfur (Log(Mn/S)) from XRF-scanning. Note long-term increasing trend after ~1 Ma related to compaction and associated changes in pore water content in the upper ~100 m of the sediment succession. **C.** Uranium concentration estimates (ppm) from shipboard spectral gamma ray data[18-19]. Continuous wavelet power spectrum computed with Morlet basis function in PAST4.10[20]; black line indicates cone of influence; dotted lines indicate 23, 41, 100 kyr periods. REDFIT spectral analyses[21] were performed for time intervals: 0.95-0.4, 1.6-0.95, 0.9-0.4, 1.2-0.9, and 1.6-1.2 Ma. Confidence intervals of 99%, 95% and 90% are given as blue, orange and green lines.

A. RABD660

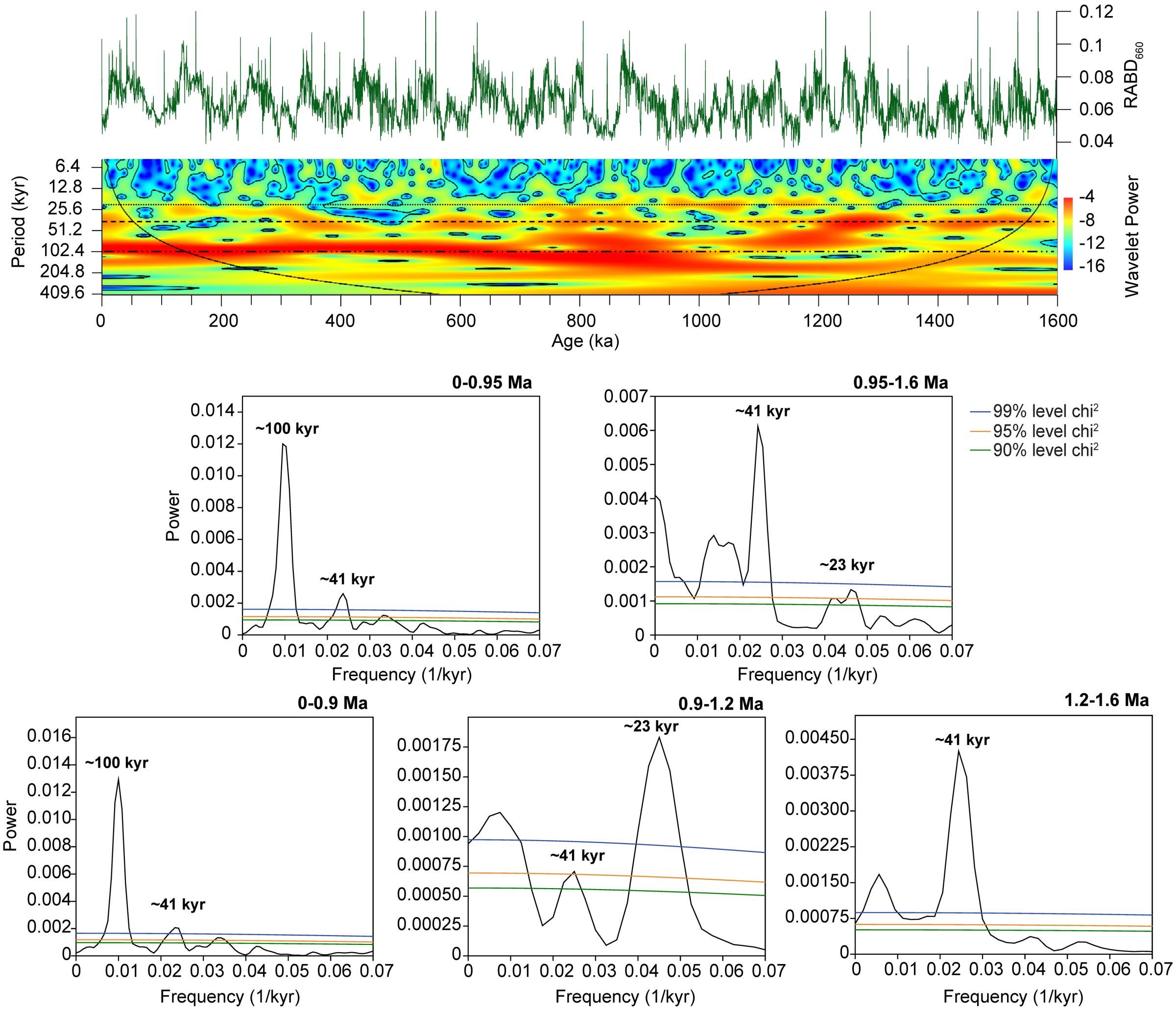

0.12
0.1
0.08
0.06
0.04
RABD660
6.4
12.8
25.6
51.2
102.4
204.8
409.6
Period (kyr)
-4
-8
-12
-16
Wavelet Power
0
200
400
600
800
1000
1200
1400
1600
Age (ka)
0-0.95 Ma
~100 kyr
~41 kyr
Power
Frequency (1/kyr)
0.95-1.6 Ma
~41 kyr
~23 kyr
99% level chi2
95% level chi2
90% level chi2
0-0.9 Ma
~100 kyr
~41 kyr
0.9-1.2 Ma
~23 kyr
~41 kyr
1.2-1.6 Ma
~41 kyr

B. Log (Mn/S)

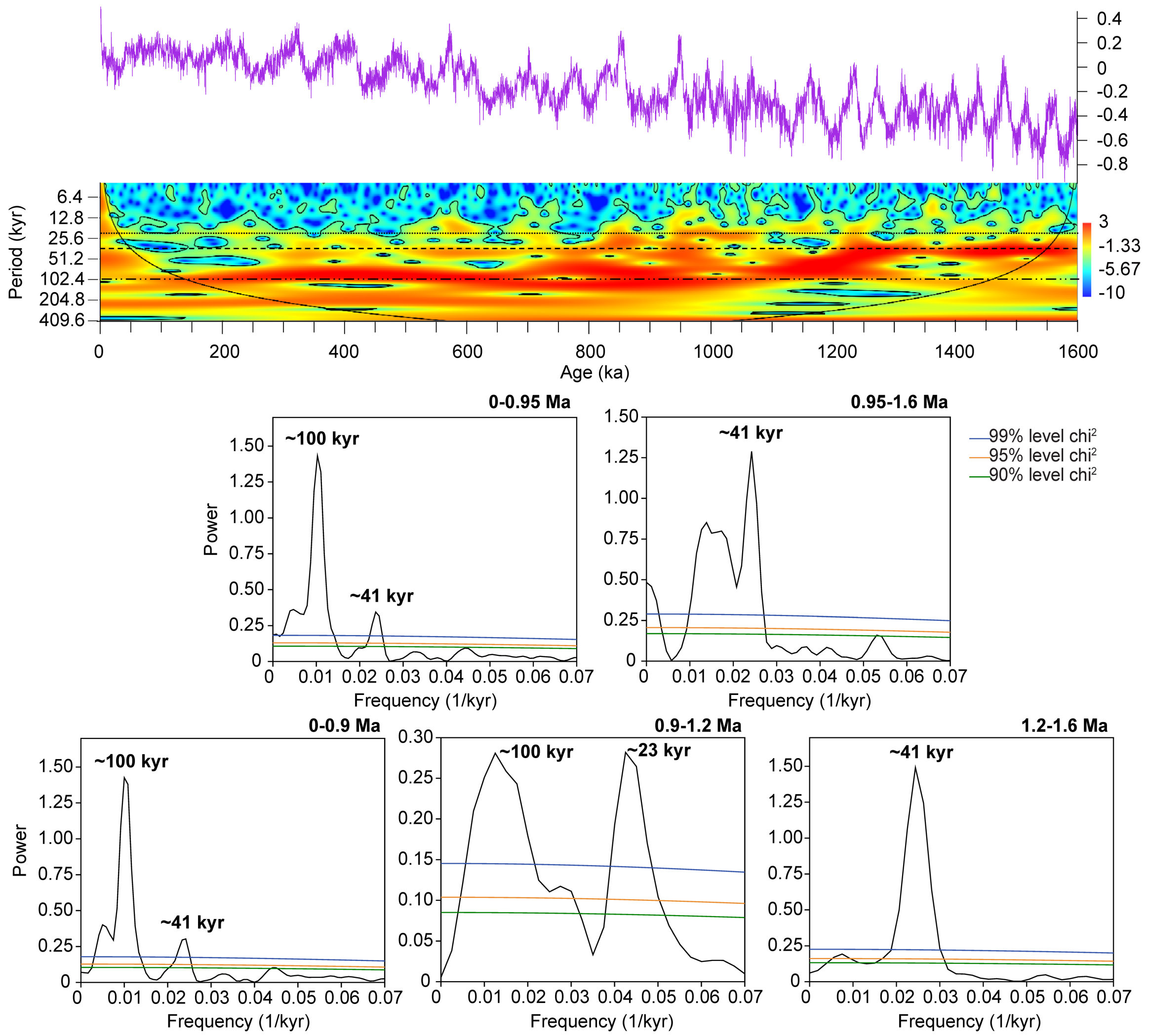

Supplementary Figure S5. Wavelet and spectral analyses of U1483 $RABD_{660}$ and Log(Mn/S) over the past 1.6 Myr. **A.** $RABD_{660}$, relative absorption band depth at 660 nm (proxy for chlorin concentration) from shipboard reflectance spectroscopy[18]. **B.** Logarithmic ratio of manganese and sulfur (Log(Mn/S)) from XRF-scanning. Note long-term increasing trend after ~1 Ma related to compaction and associated changes in pore water content in the upper ~100 m of the sediment succession. Continuous wavelet power spectrum computed with Morlet basis function in PAST4.10[20]; black line indicates cone of influence; dotted lines indicate 23, 41, 100 kyr periods. REDFIT spectral analyses[21] were performed for time intervals: 0.95-0, 1.6-0.95, 0.9-0, 1.2-0.9, and 1.6-1.2 Ma. Confidence intervals of 99%, 95% and 90% are given as blue, orange and green lines.

Riverine terrigenous discharge

We used the sum of XRF-scanner K-alpha area counts per second (cps) of the typical clay mineral-derived elements aluminum (Al), potassium (K), Fe and titanium (Ti), abbreviated as Terr, as a proxy for the terrigenous sediment-component, mainly originating from the Australian continent via riverine transport into the Timor Sea. Comparison of XRF-scanner area cps counts, measured with the same settings on the same instrument in two neighboring cores, to absolute elemental concentrations based on 20 fused beads XRF analyses of decarbonatized sediment indicated clear linear relationships with R^2 of 0.74 for Al, 0.93 for K, 0.98 for Fe and 0.98 for Ti[22]. We did not include silicon (Si) and zirconium (Zr) in Terr, since these elements commonly occur in quartz and zircon grains, which are subject to sorting processes during transport and may be wind-transported during glacials. This is also partly the case for Ti, which is included in Terr, but does not significantly influence the record due to its low area cps values and small deviation from other terrigenous elements. The Si record nevertheless exhibits high similarity to the Terr record, suggesting that the contribution of windblown quartz grains and biogenic opal to the total Si record is low. Rubidium (Rb), a common replacement of K in the clay mineral illite, exhibits an almost identical trend to that of the other clay mineral derived elements (Supplementary Figure S6). However, Rb was not included in Terr, since it is the only clay-derived element that was not measured with the 10 kV setting of the XRF scanner and, thus, area cps counts are not comparable. We normalized Terr using its log-ratio to calcium (Ca) that is mainly derived from the biogenic carbonate of marine plankton. This approach is commonly used in carbonate-rich environments, where no indication of significant changes in carbonate dissolution or in carbonate productivity are detected[3,23-26]. We, therefore, calculate the logarithmic ratio of clay-mineral bound terrigenous elements normalized to Ca using the formula:

Log((Al_area cps + K_area cps + Fe_area cps + Ti_area cps)/Ca_area cps), abbreviated as Log(Terr/Ca)

We also note that the orbital-scale variability of the riverine sediment discharge proxies covary with that of the Log(Al/K) ratio, which is associated with changes in chemical weathering conditions within the catchment areas of the northwestern Australian rivers (Supplementary Figure S7). The K-enriched clay mineral illite is generally a weathering product in temperate and arid climates, where physical weathering is dominant. By contrast, Al-rich kaolinite is usually a product of chemical weathering in humid climates[27-29]. Accordingly, high Log(Al/K) values correlate with higher rates of soil weathering during

periods of increased precipitation in the source area and low Log(Al/K) values correspond to lower weathering rates associated with generally dryer conditions and/or higher erosion rates under higher rainfall seasonality.

Carbonate-free basis normalization [element abundance x 100 / (100 − $CaCO_3$)] was additionally used to evaluate the influence of variations in carbonate accumulation rates on terrigenous elemental concentrations. Despite being influenced by the weathering regime we consider K as a representative terrigenous element, since K is a key component in illite-rich clay mineral assemblages derived from NW Australian rivers[30] and XRF-scanner measurements of K (in contrast to Al) are not influenced by the water content of sediment cores[16]. Concentrations of K in sediment cores were calculated from the shipboard spectral gamma ray data[18-19], then used for calibration of the high-resolution XRF-scanner derived records (Supplementary Figures S9-S10). XRF scanner-derived Ca area counts were transferred into $CaCO_3$ weight percentages using high-resolution carbonate concentration data from Core MD01-2378, located 0.8 nmi northwest of Site U1483[5] (Supplementary Figure S9). Carbonate-free basis normalized K data exhibit the same orbital scale (precession and obliquity) variability as Log (Terr/Ca) and K concentrations in bulk sediment, indicating that variability is not driven by changes in carbonate accumulation rates.

Differences in the abundance of Fe, Si, Al, K, Rb, Ti and Zr related to composition, grain size and transport pathway of terrigenous particles[31] are recognizable in our XRF-scanner records (Supplementary Figures S6 and S8). Potassium and Al occur preferentially in river-transported fine-grained clay, whereas Zr, Ti and Fe are usually enriched in larger and/or heavier sediment grains. Titanium and Zr are the main components of heavy minerals such as rutile and zircon, which are subject to sorting and preferential settling close to river mouths and on the continental shelf. At more southwesterly locations along the NW Australian margin, a substantial proportion of the Zr, Ti and Fe content is of aeolian origin[22,32-34]. However, aeolian dust represents only a minor sedimentary component at the location of Site U1483.

The shipboard natural gamma ray core logging (NGR) system of the JOIDES Resolution allows measurement of the intensity of gamma radiation at 1024 energy levels between 0 and 3000 keV, thus, enabling quantitative estimates of the elemental K, U and thorium (Th) concentrations in the sediment cores from the shipboard energy spectra[19, 35]. The potassium concentrations from spectral gamma ray measurements correlate significantly ($R = 0.76$) with

the XRF scanner-derived K_area cps (Supplementary Figure S9). Spectral characteristics of spectral gamma ray derived estimates of K concentrations and Log (Terr/Ca) are almost identical (Supplementary Figures S11 and S12), suggesting that illite is a representative component of clay mineral assemblages derived from the NW Australian source area.

Fluctuating sea levels may determine the discharge of terrigenous sediments offshore major river and delta systems with predominant deposition of marine carbonates during sea level highstands and increased input of fluvial sediment load during sea level lowstands, when the coastline was more proximal and rivers drained more directly over the shelf edge[30]. However, the main increase and maximum in terrigenous discharge along the NW Australian margin occurred in the late stage of glacial terminations and during interglacials, when the sea level was rising or close to maximum. The occurrence of peak monsoonal sediment discharge during interglacials, when the distance between Site U1483 and the adjacent river mouths was at a maximum, clearly excludes proximity to the coastline as the main driver of terrigenous sediment accumulation at this site. The influence of glacial interglacial sea-level changes on the XRF-scanner derived sediment composition at Site U1483 over the last 0.41 Myr, previously evaluated using Ca-normalized terrigenous elemental data, was not found discernible[3]. We relate the lack of a sea level imprint on the terrigenous sediment discharge at Site U1483 to the relatively far distance of the site location to the coastline even during glacial sea level lowstands (Figure 1) and to the efficient transport and direct delivery of the sediment load to the Timor Sea without the development of huge flood plains and delta systems. Increases in terrigenous sediment discharge are, thus, directly associated with erosion and runoff by intensified monsoonal precipitation in the catchment area.

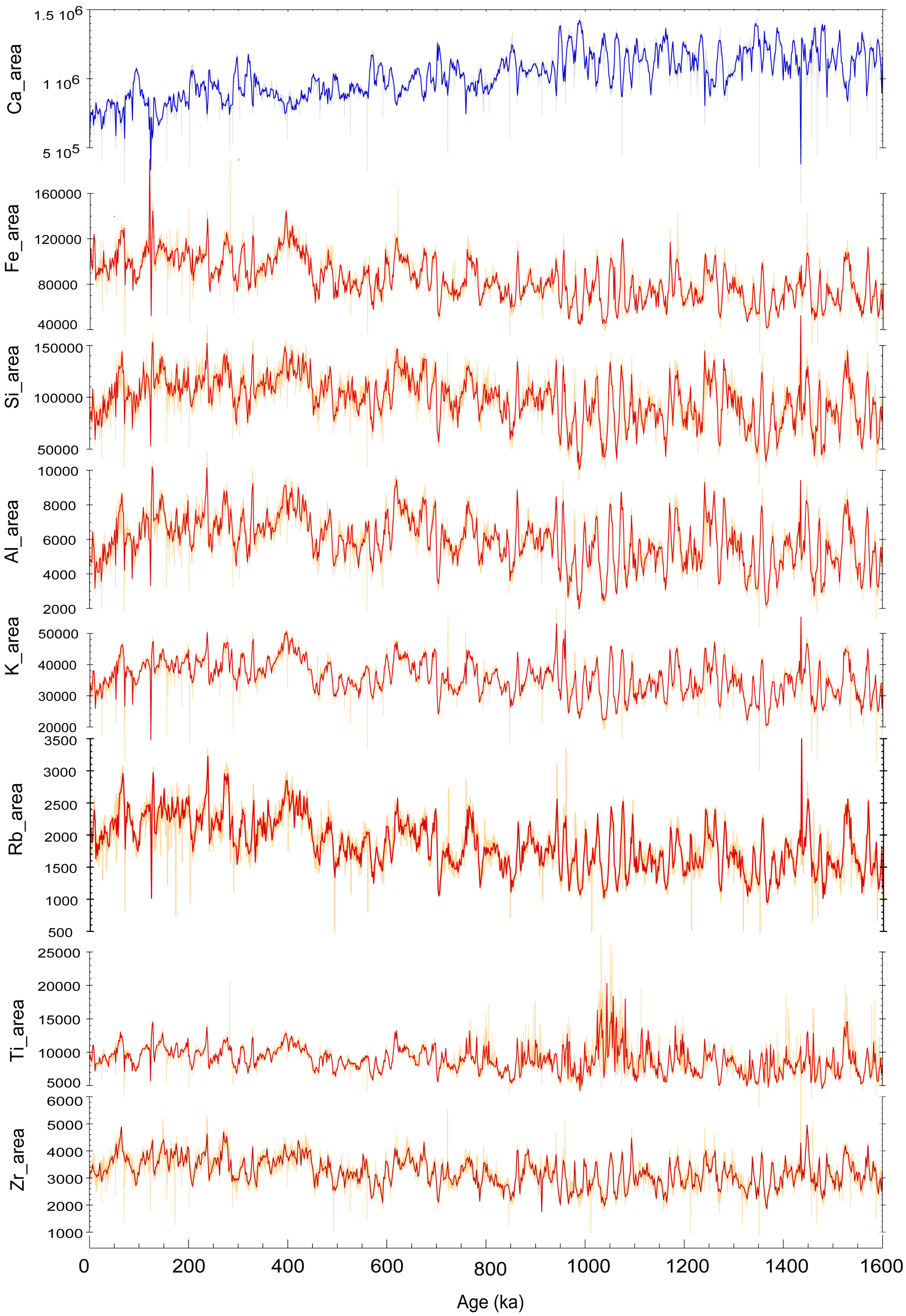

Supplementary Figure S6. U1483 XRF-scanner area counts per second (cps) for key elements of biogenic carbonate (Ca) and terrigenous discharge (Fe, Si, Al, K, Rb, Ti and Zr) over the past 1.6 Myr. Thin lines show raw counts, thick lines show Stineman interpolations (interpolate function in Kaleidagraph 5.0 software). Note almost identical Fe, Si, Al, K and Rb plots, whereas Ti and Zr plots show substantial differences.

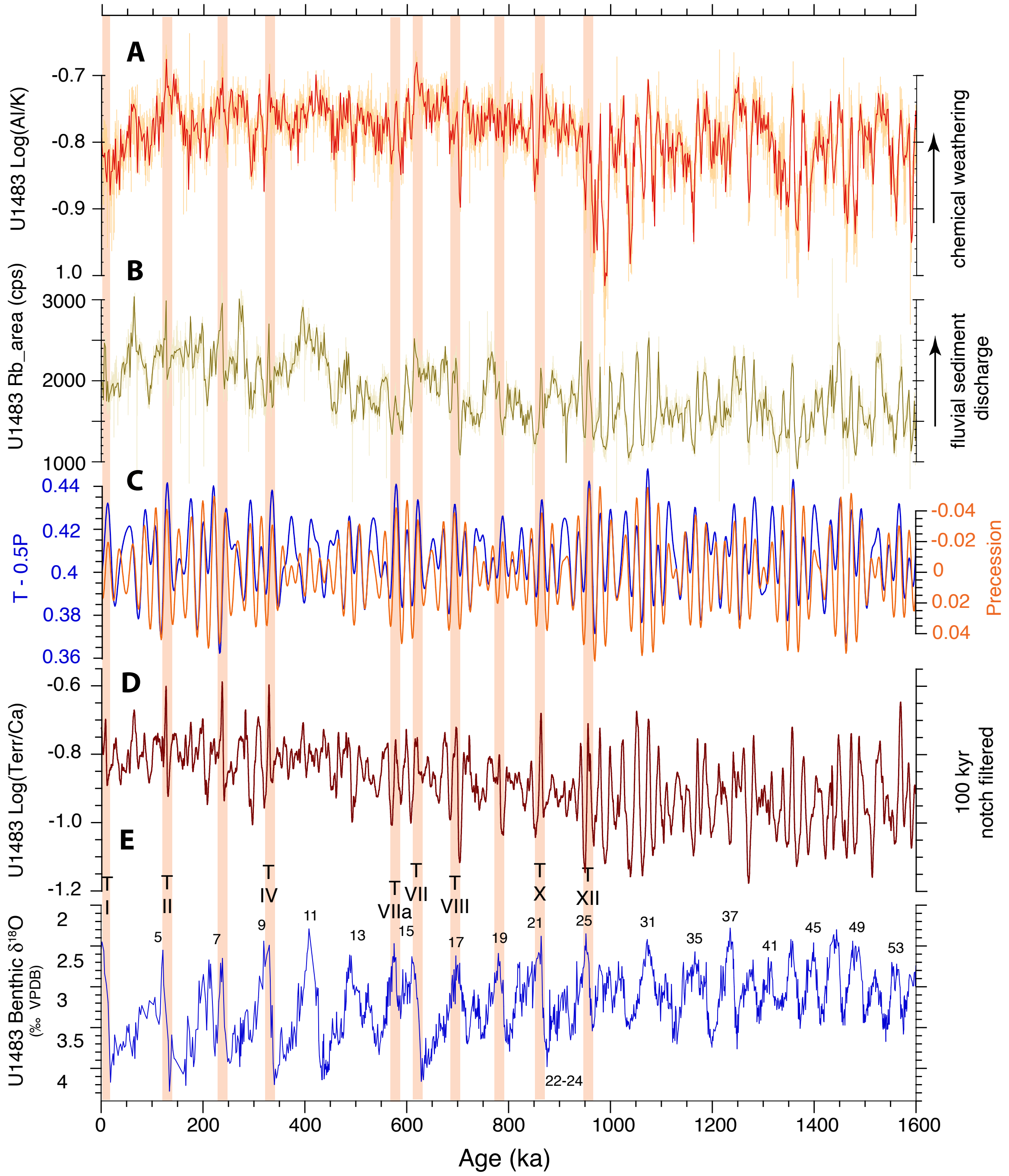

Supplementary Figure S7. Concurrent increase in chemical weathering and discharge at the onset of warm interglacials (marked with pink shading) and during precession minima/obliquity maxima (T-0.5P maxima) after 950 ka. **A.** Chemical weathering indicator Log(Al/K) from Site U1483. **B.** Riverine sediment discharge proxy Rb_area counts per second (cps) from Site U1483. **C.** Orbital configuration[36]. **D.** Riverine sediment discharge proxy Log(Terr/Ca) from Site U1483. **E.** U1483 benthic foraminiferal $\delta^{18}O$ with data from 0 to 410 ka from ref. 3. T: Termination.

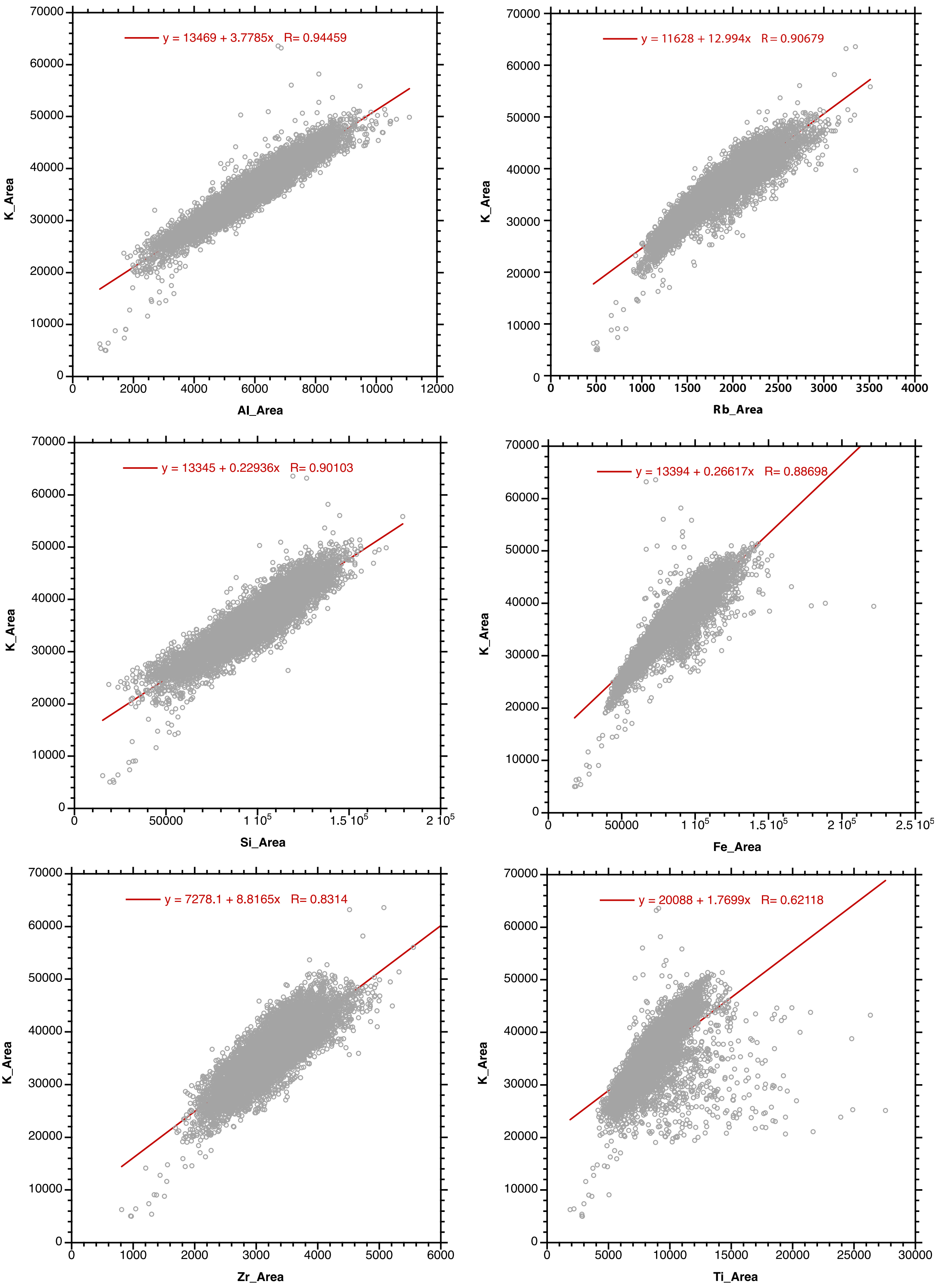

Supplementary Figure S8. Crossplots of U1483 XRF-scanner derived K_area counts per second (cps) against other terrigenous elements. Note strong correlation of K to other clay-mineral (illite) elements (Al, Rb, Si, and Fe). Zr and Ti are grain-size related and exhibit weaker correlations.

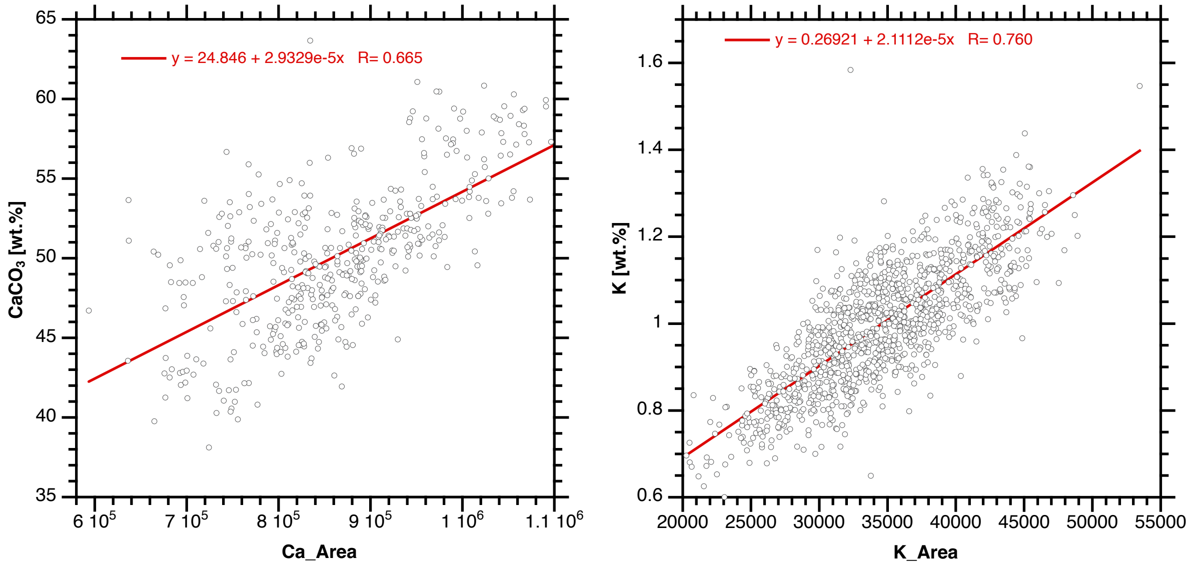

Supplementary Figure S9. Regression plots of U1483 Ca_area counts per second (cps) against $CaCO_3$ weight percent and of U1483 K_area cps against bulk sediment K weight percent. $CaCO_3$ weight percent and K weight percent data are derived from discrete measurements with a LECO combustion-analyzer[5] for $CaCO_3$ and from spectral gamma ray data for K[19].

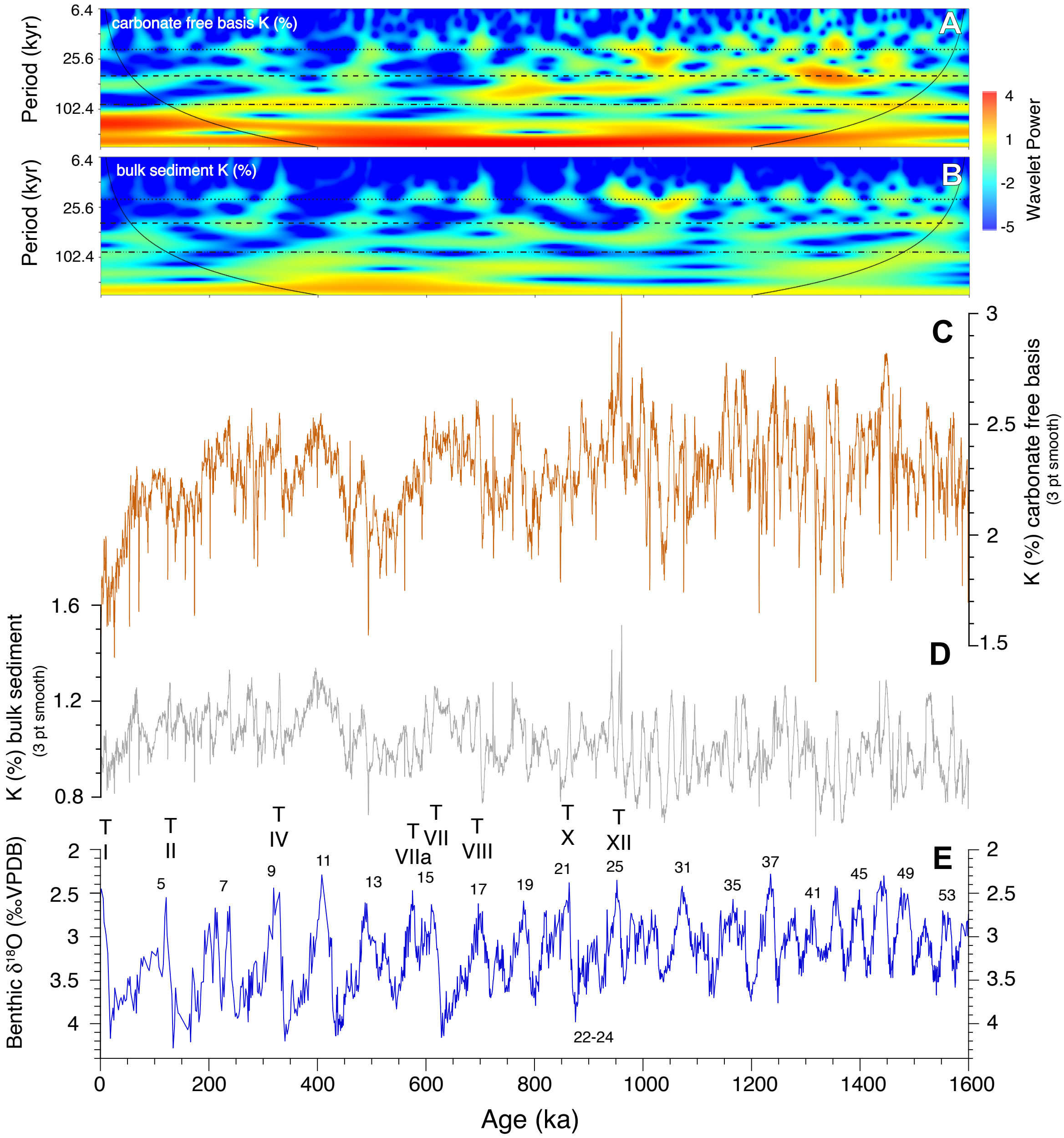

Supplementary Figure S10. Comparison of U1483 XRF-scanner derived bulk sediment and carbonate free basis potassium (K) concentrations. **A.** Wavelet power spectrum of carbonate-free basis K concentrations. **B.** Wavelet power spectrum of bulk sediment K concentrations. **C.** Three point moving average of carbonate free basis K concentrations. **D.** Potassium concentrations calculated from shipboard spectral gamma ray data[8,13], used for calibration of XRF-scanner derived records. **E.** U1483 benthic foraminiferal $\delta^{18}O$ with data from 0 to 410 ka from ref. 3. Continuous wavelet power spectrum in A-B computed with Morlet basis function in PAST4.10[20]; black line indicates cone of influence; dotted lines in wavelet power spectrum indicate 23, 41, 100 kyr periods. T: Termination.

A. Log(Terr/Ca)

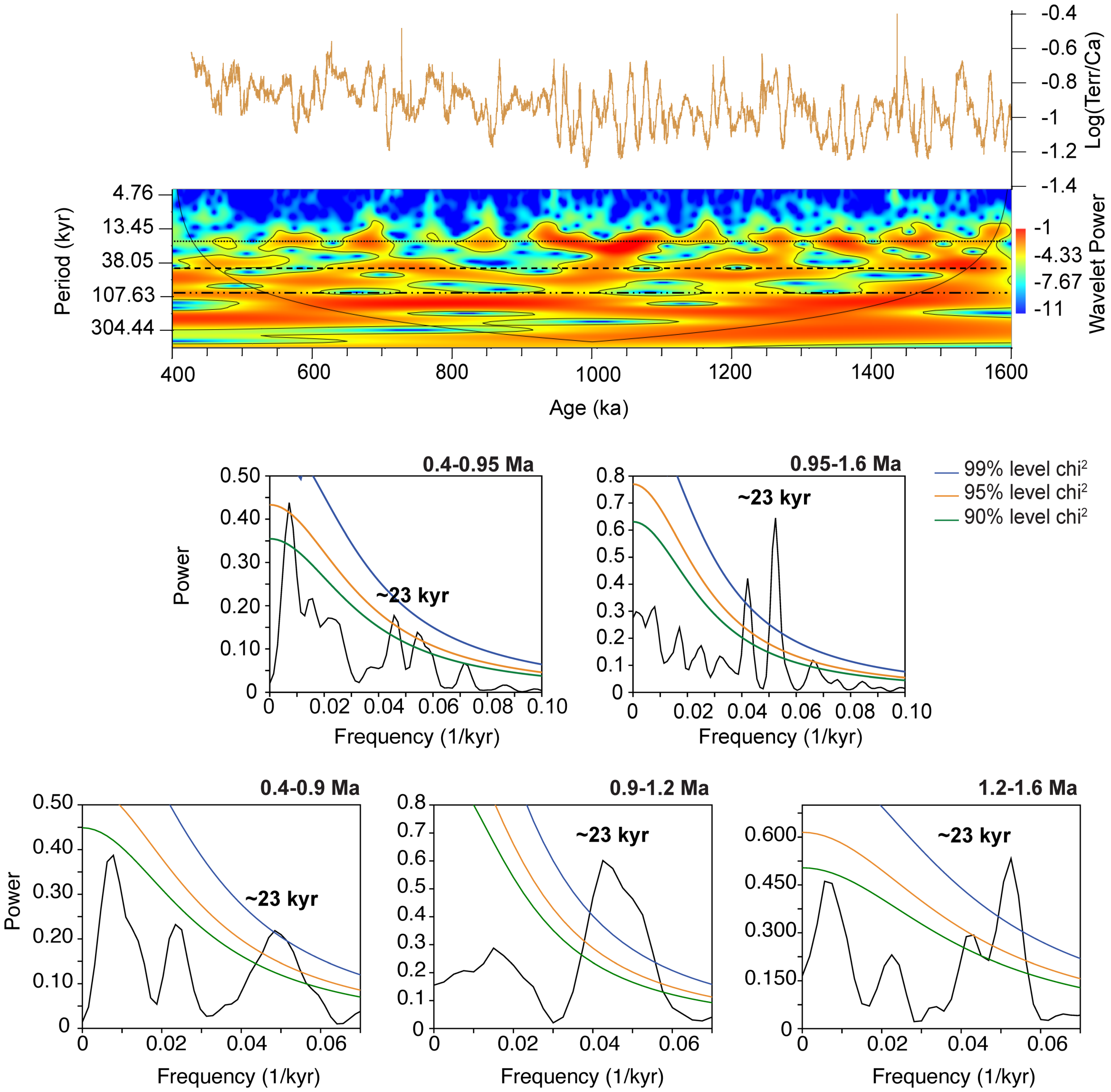
Log(Terr/Ca)
Period (kyr)
Wavelet Power
Age (ka)
0.4-0.95 Ma
0.95-1.6 Ma
~23 kyr
Power
Frequency (1/kyr)
99% level chi2
95% level chi2
90% level chi2
0.4-0.9 Ma
0.9-1.2 Ma
1.2-1.6 Ma

B. K (concentrations)

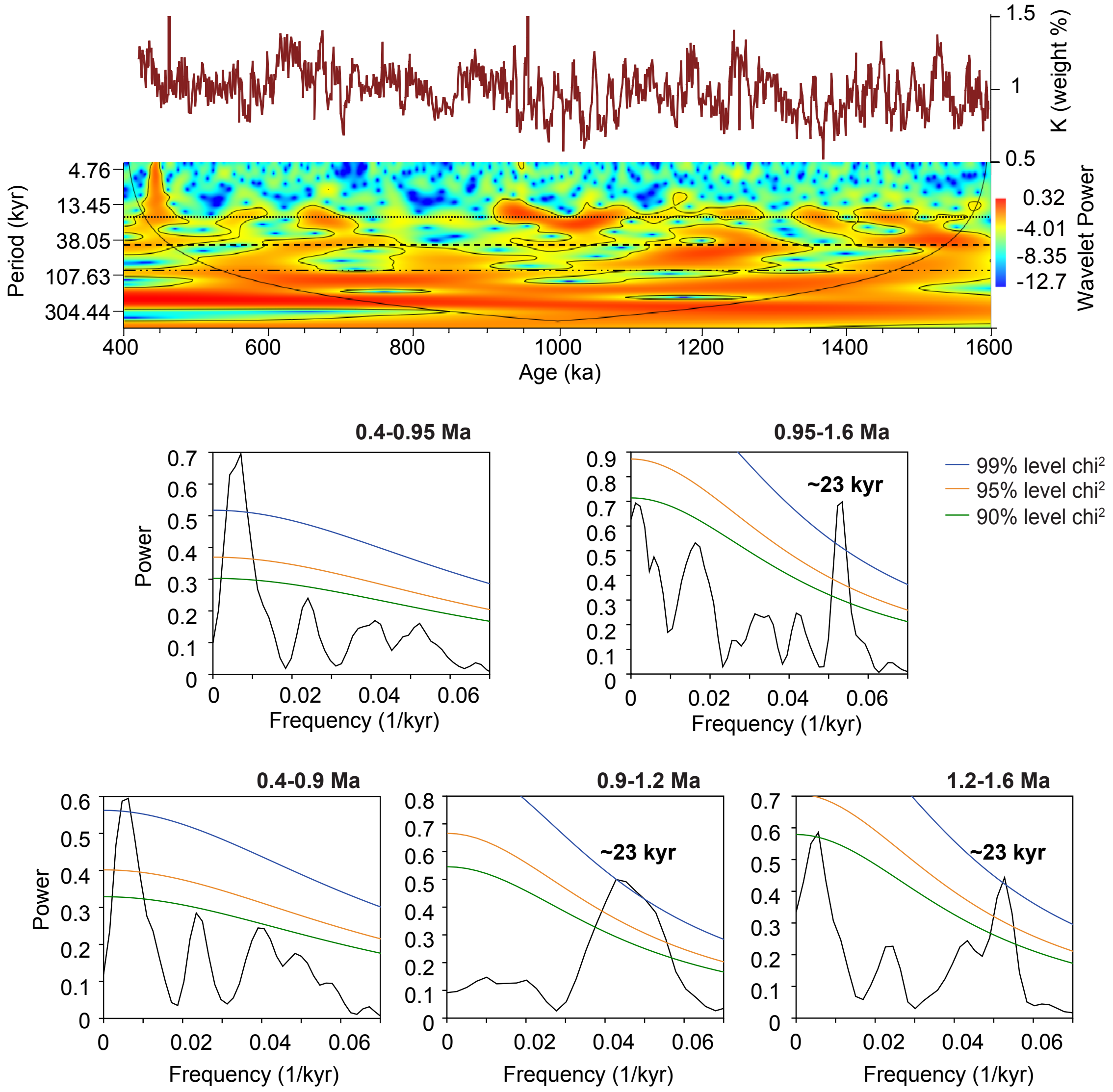

Supplementary Figure S11. Wavelet and spectral analyses of U1483 Log(Terr/Ca) and K (weight %) for time interval 1.6 to 0.4 Ma. **A.** Logarithmic ratio of Log(Terr/Ca) from XRF-scanning. **B.** Potassium concentrations calculated from shipboard spectral gamma ray data[18,19], used for calibration of XRF-scanner derived records. Continuous wavelet power spectrum computed with Morlet basis function in PAST 4.10[20]; black line indicates cone of influence; dotted lines indicate 23, 41, 100 kyr periods. REDFIT spectral analyses[21] were performed for time intervals: 0.95-0.4, 1.6-0.95, 0.9-0.4, 1.2-0.9, and 1.6-1.2 Ma. Confidence intervals of 99%, 95% and 90% are given as blue, orange and green lines.

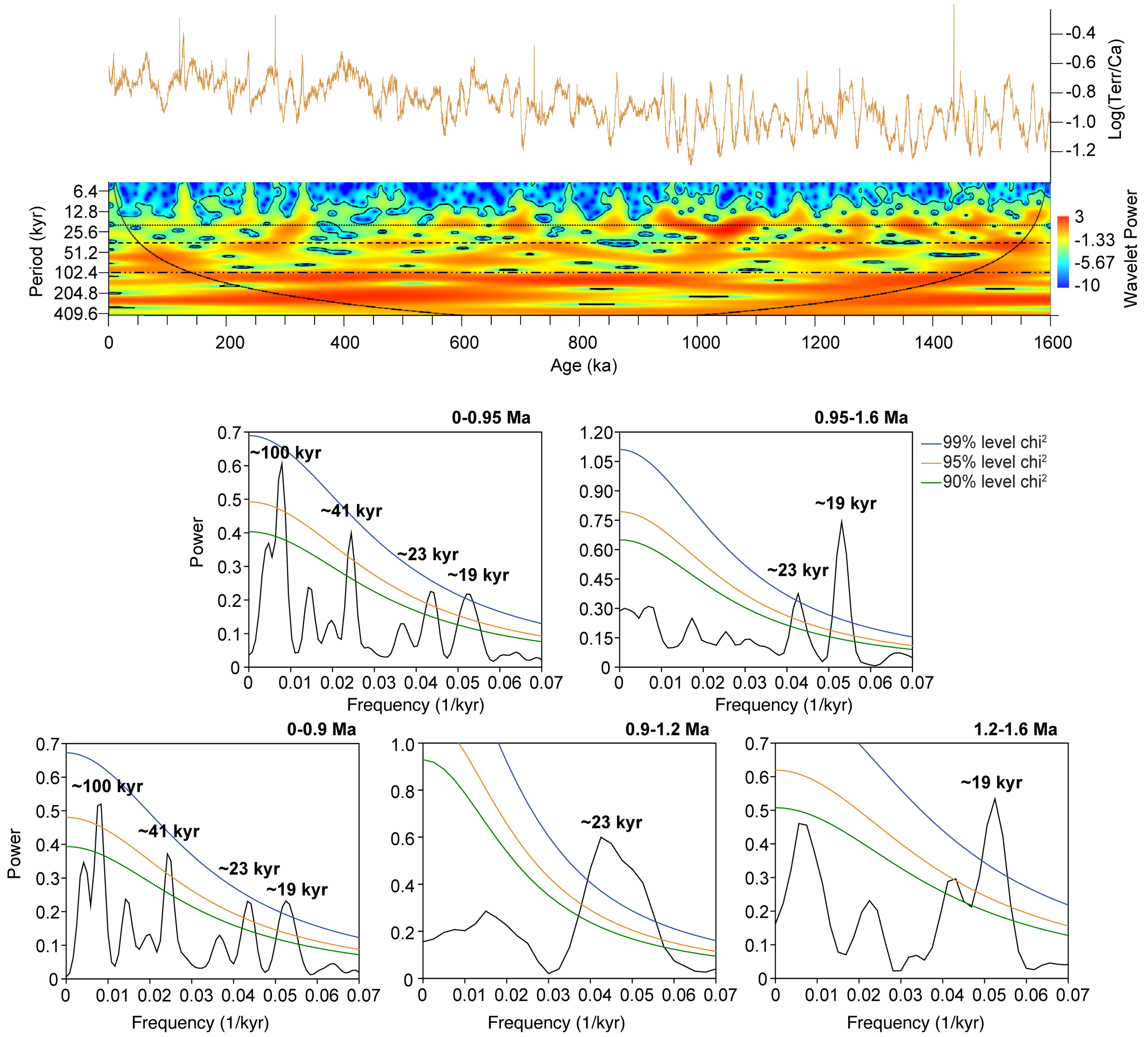

Supplementary Figure S12. Wavelet and spectral analyses of (Log Terr/Ca) over past 1.6 Myr. Continuous wavelet power spectrum computed with Morlet basis function in PAST4.10[20]; black line indicates cone of influence; dotted lines indicate 23, 41, 100 kyr periods. REDFIT spectral analyses[21] were performed for time intervals: 0.95-0, 1.6-0.95, 0.9-0, 1.2-0.9, and 1.6-1.2 Ma. Confidence intervals of 99%, 95% and 90% are given as blue, orange and green lines.

Cross spectral analysis

Cross-spectral analyses were performed with the Blackman-Tukey approach using AnalySeries version 2.08[37]. All data are interpolated to a constant time step close to the actual data resolution, and pre-treated by removal of linear trend and pre-whitening. All spectra use a Bartlett window and a 30% lag. The bandwidth varies between 0.0056 and 0.0117. Non-zero coherence is higher than ~0.385 (Supplementary Table S2, Supplementary Figures S13-15).

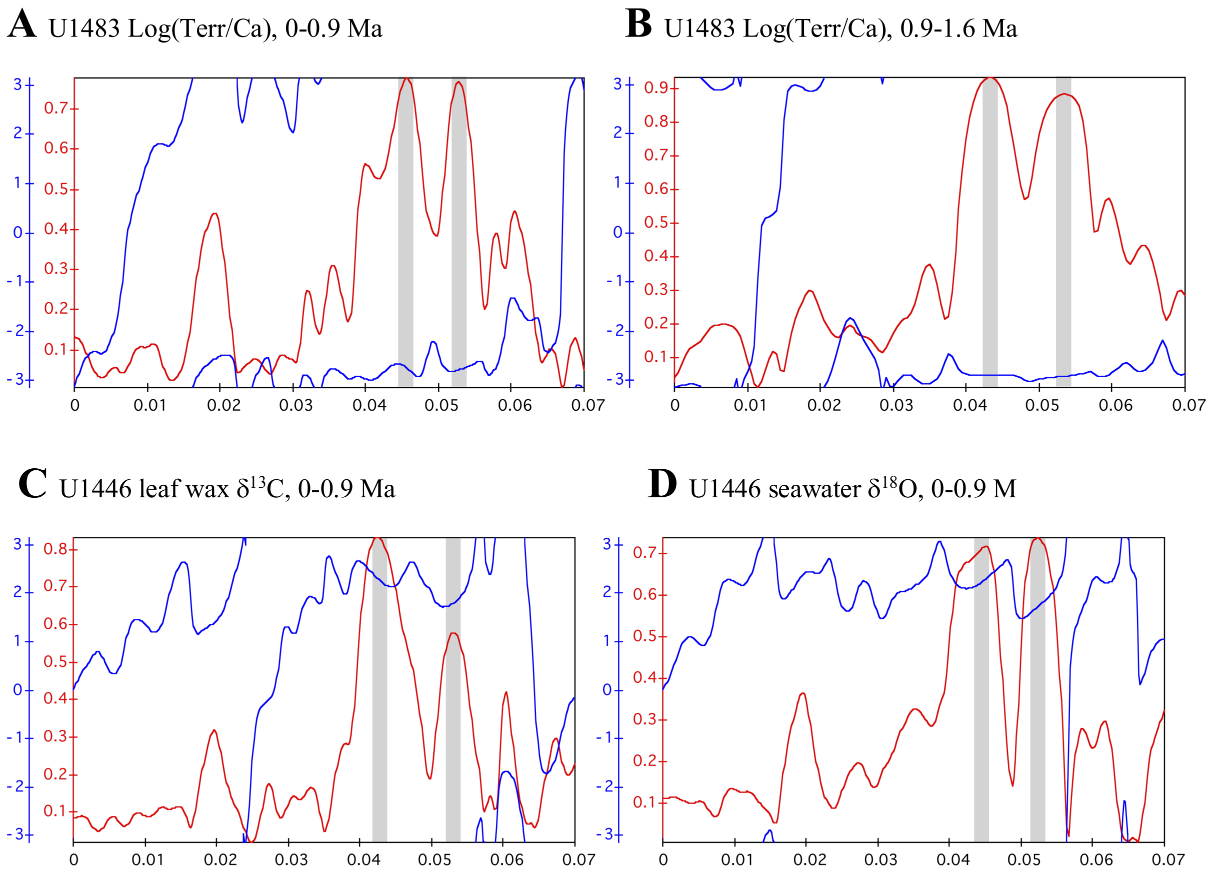

Supplementary Figure S13. A-B. Blackman-Tukey cross spectral analyses of precession parameter with U1483 Australian Summer Monsoon proxy record (Log(Terr/Ca)) for time intervals: 0-0.9 Ma and 0.9-1.6 Ma. **C.-D.** Blackman-Tukey cross spectral analysis of precession parameter with Indian Monsoon proxies from Bay of Bengal Site U1446[38] for time interval: 0-0.9 Ma. Note: y-axes represent coherence (red) and phase (in radians, blue), x-axes denote frequency (1/kyr). Gray shading marks precessional frequencies with high coherence.

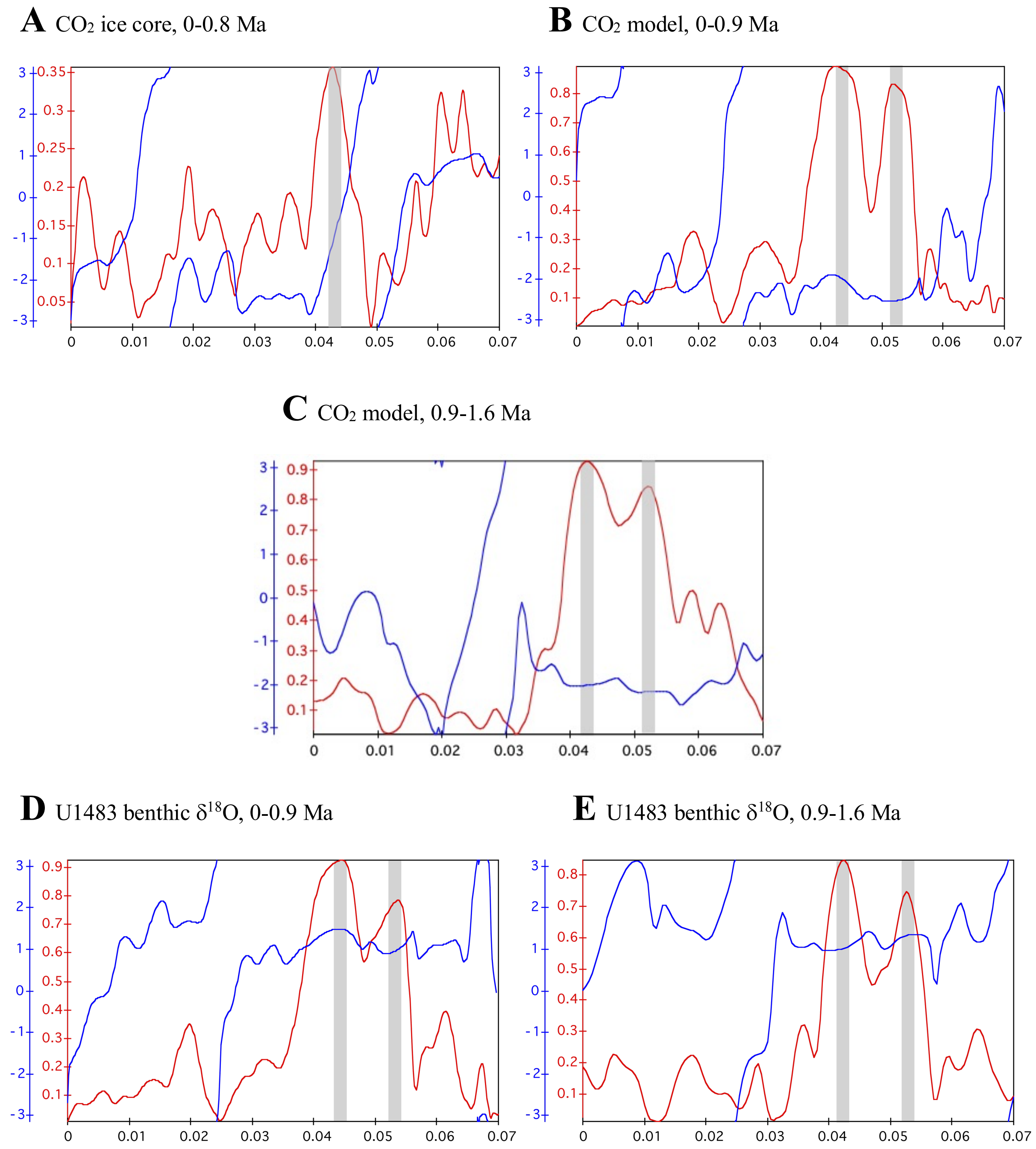

Supplementary Figure S14 A.-E. Blackman-Tukey cross spectral analysis of precession parameter with internal feedback/boundary conditions. **A.** Atmospheric pCO_2 from ice core (0-0.8 Ma)[39]. **B-C.** Model pCO_2 data[40] for time intervals: 0-0.9 Ma and 0.9-1.6 Ma. **D-E.** Ice volume (benthic foraminiferal $\delta^{18}O$ at Site U1483) for time intervals: 0-0.9 Ma and 0.9-1.6 Ma. Note: y-axes represent coherence (red) and phase (in radians, blue), x-axes denote frequency (1/kyr). Gray shading marks precessional frequencies with high coherence.

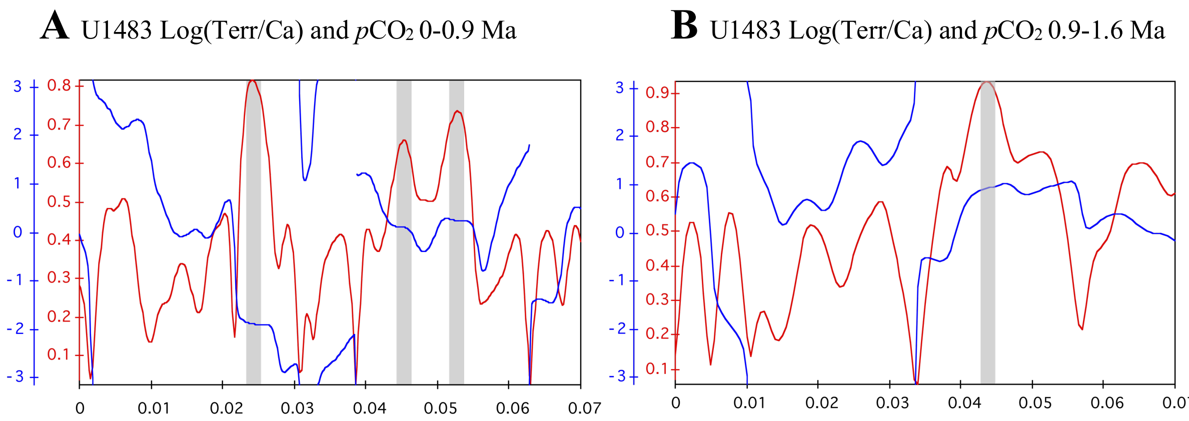

Supplementary Figure S15 Blackman-Tukey cross spectral analysis of U1483 Log(Terr/Ca) with atmospheric pCO_2 model data[40]. **A.** For time intervals: 0-0.9 Ma. **B.** For time interval 0.9-1.6 Ma. Note: y-axes represent coherence (red) and phase (in radians, blue), x-axes denote frequency (1/kyr). Gray shading marks obliquity and precessional frequencies with high coherence.

Supplementary Table S2. Coherency and phase relationship of proxy data relative to orbital parameters. Higher and lower phase shows error limits in radians. NH insolation = Northern Hemisphere insolation at 65°N on 21st June; ENSO = insolation at 25°S on 10th September; SITIG 21 Jun = summer inter-tropical insolation gradient (insolation at 23°N minus insolation at 23°S) on 21st June; ice min = $\delta^{18}O$ minimum in the LR04 $\delta^{18}O$ stack[2]. U1446 $\delta^{18}O_{sw}$ and leaf wax $\delta^{13}C$ records are from Site U1446[38]; pCO_2 ice core (0-0.8 Ma)[39,41-45]; pCO_2 model = reconstructed CO_2 from ref. 40.

Time intervals	Proxy	Coherency k	Phase (rad) higher	Phase (rad) middle	Phase (rad) lower	Phase (deg)	±limit (deg)	Phase (kyr)	±limit (kyr)	Phase lag to -P (deg)
**0-0.9 Ma**	NH insolation	1		-3.14		-180	0	-11.5	0.0	0
	September insolation 25°S	1		-1.76		-101	0	-6.4	0.0	79
	SITIG 21 June	1		-3.14		-180	0	-11.5	0.0	0
	U1483 Log(Terr/Ca)	0.6	-2.26	-2.7	-3.17	-155	26	-9.9	1.7	25
	U1483 $\delta^{18}O_{ben}$ min	0.92	1.62	1.47	1.32	84	9	5.4	0.5	84
	ice min	0.88	1.46	1.28	1.09	73	11	4.7	0.7	73
	U1446 $\delta^{18}O_{sw}$	0.69	2.5	2.12	1.77	121	21	7.8	1.3	121
	U1446 leaf wax $\delta^{13}C$	0.8	2.46	2.19	1.95	125	15	8.0	0.9	125
	pCO_2 ice core (0-0.8 Ma)	0.8	-1.55	-1.83	-2.08	-105	15	-6.7	1.0	75
	pCO_2 model	0.89	-1.79	-1.99	-2.18	-114	11	-7.3	0.7	66
**0.9-1.6 Ma**	NH insolation	1		-3.14		-180	0	-11.5	0.0	0
	September insolation 25°S	1		-1.76		-101	0	-6.4	0.0	79
	SITIG 21 June	1		-3.14		-180	0	-11.5	0.0	0
	U1483 Log(Terr/Ca)	0.94	-2.5	-2.63	-2.7	-151	6	-9.6	0.4	29
	U1483 $\delta^{18}O_{ben}$ min	0.81	1.36	1.09	0.85	62	15	4.0	0.9	62
	ice min	0.9	1.38	1.19	1.03	68	10	4.4	0.6	68
	pCO_2 model	0.91	-1.83	-2	-2.16	-115	9	-7.3	0.6	65

Supplementary Material 3: Sea surface temperature trends between 1.2 and 0.8 Ma

When compiling latitudinal sea surface temperature (SST) records, our aim was to select sites that were representative for the evolution of SST gradients in the Northern (NH) and Southern Hemispheres (SH) (Supplementary Table S3, Supplementary Figure S16). We excluded high-productivity equatorial and coastal upwelling regions along the Pacific and Atlantic eastern margins such as Sites 1012, 1020 and 1082-1084, as the evolution of regional SST at these locations is mainly driven by regional processes involving complex current dynamics that are not coupled directly to high-latitude climate evolution[46-47]. In the SH Pacific Ocean, we selected the Tasman Sea Site 593 and excluded Site 1125 at comparable latitude on the Chatham Rise, east of New Zealand[47] because Site 1125 is in a region of high surface productivity, as shown by the abundance of siliceous microfossils in Pleistocene sediments[48]. By contrast, Site 593 is not characterized by high surface productivity and is more directly influenced by Antarctic Intermediate Water[49].

In Figure 4, we selected the NH high latitude Site 882 in the subarctic Pacific Ocean[50], because the evolution of SST between 1200 and 800 ka in the North Atlantic exhibits contrasting trends with simultaneous cooling and warming along the northwestern and southeastern margins (respectively) of the North Atlantic Current during MIS 28 (~995 ka)[51]. The shift from 41 to ~100 kyr cyclicity was also diachronous in these two regions[51]. The later shift to the southeast of the North Atlantic Current was attributed to the gradual southward spreading influence of NH ice sheets[51]. By contrast, the impact of high latitude climate cooling and ice sheet growth on North Pacific Ocean SST appears more straightforward during this interval. In the SH, we selected Site U1090, located in the subantarctic Atlantic, as the SST record from this site exhibits strong similarities to the spectral characteristics of Antarctic ice core climate records[50].

Both records from Sites 882 and 1090 are based on the original U^{K}_{37} index, which exhibits a more robust relationship to annual mean SSTs than U^{K}_{37}' at high latitudes[52-53]. Moreover, the U^{K}_{37} index includes the tetra-unsaturated alkenone (C37:4), which is now regarded as a sea ice proxy[54], thus providing additional information on the regional variability of fall and winter climate conditions at high latitudes. This index may, however, bias SST estimates to colder values in contrast to U^{K}_{37}', which preferentially record temperature during the favourable spring and summer growth period for alkenone producing phytoplankton. This bias may explain the strikingly low U^{K}_{37}-based SST estimates at Site 882, likely associated with an increase in seasonal sea ice between ~1 and ~0.95 Ma in the Bering Sea[55].

Supplementary Table S3: Data sources for Supplementary Figure S16 (latitudinal sea surface temperature trends between 1.2 and 0.8 Ma). Age models: specified proxy tuned to LR04[2], Sh677[56] or BL91[57].

Site	Latitude	Longitude	Water depth (km)	Location	Original age model	Mean resolution (ka)	SST proxy	Reference
**ODP 982**	57°30′N	15°52′W	1.134	North Atlantic Ocean	Benthic $\delta^{18}O$ (LR04)	5	U^{K}_{37}'	Lawrence et al. (2009)[58]
**ODP 882**	50°21′N	167°35′W	3.244	Subarctic NW Pacific	GRAPE (BL91)	6.5	U^{K}_{37}	Martinez-Garcia et al. (2010)[50]
**ODP 1146**	19°27′N	116°16′E	2.092	South China Sea	Benthic $\delta^{18}O$ (LR04)	1.7	U^{K}_{37}'	Herbert et al. (2010)[59]
**ODP 722**	16°37′N	59°48′E	2.022	Arabian Sea	Benthic $\delta^{18}O$ (LR04)	1.9	U^{K}_{37}'	Herbert et al. (2010)[59]
**MD97-2140**	2°02′N	141°46′E	2.547	Western Pacific	Benthic $\delta^{18}O$ (Sh677)	4.9	Mg/Ca in *G. ruber*	de Garidel-Thoron et al. (2005)[60]
**MD06-3018**	23°00′S	166°09′E	2.47	Coral Sea	Benthic $\delta^{18}O$ (LR04)	5.9	Mg/Ca in *G. ruber*	Russon et al. (2010)[61]
**IODP U1460**	27°22.4867′S	112°55.4265′E	0.215	Eastern Indian Ocean	Benthic $\delta^{18}O$ (LR04)	7.9	TEX_{86}	Petrick et al. (2019)[62]
**DSDP 593**	40°30.47′S	167°40.47′E	1.05	Tasman Sea, Southwestern Pacific	Benthic $\delta^{18}O$ (LR04)	7.5	U^{K}_{37}'	McClymont et al. (2016)[63]
**ODP 1090**	42°54.8'S	8°53.9'E	3.7	Subantarctic Atlantic	Benthic $\delta^{18}O$ (LR04)	2.6	U^{K}_{37}	Martinez-Garcia et al. (2010)[50]

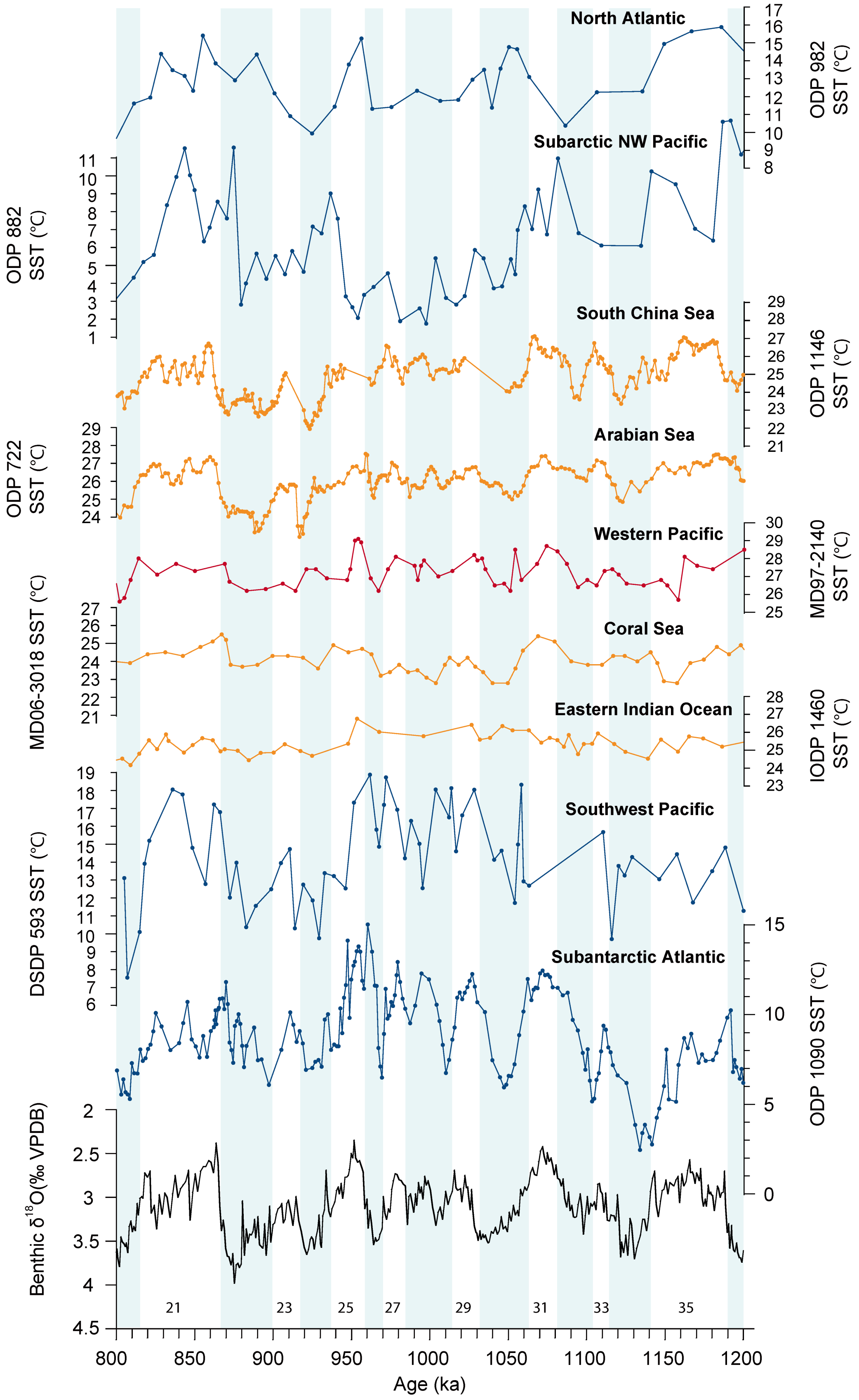

Supplementary Figure S16: Latitudinal sea surface temperature (SST) trends at representative Southern and Northern Hemisphere locations between 1.2 and 0.8 Ma. Note high-latitude Northern Hemisphere cooling in antiphase to high-latitude Southern Hemisphere warming in contrast to low SST variability at low-latitudes. Original temperature data sources are listed in Supplementary Table S3; benthic foraminiferal $\delta^{18}O$ record from Site U1483.

Supplementary Material 4: Spectral characteristics of Asian monsoonal proxy records over past 950 kyr

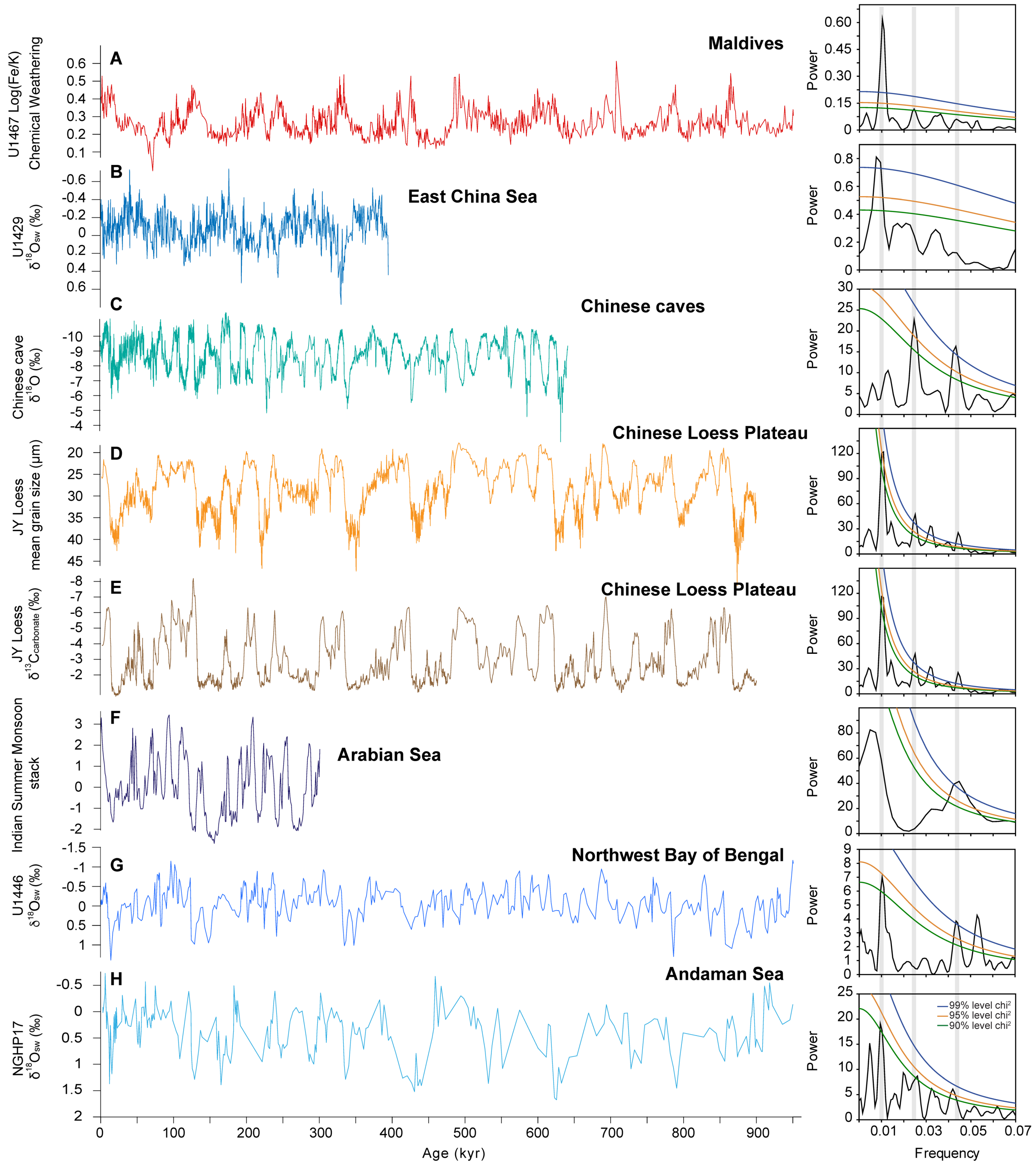

Supplementary Figure S17: Spectral characteristics of selected Asian monsoonal proxy records over last 950 kyr. **A.** Chemical weathering proxy Log(Fe/K) from Maldives Site U1467[64]. **B.** Ice volume corrected seawater oxygen isotopes ($\delta^{18}O_{sw}$) from Site U1429 as salinity/Yangtze River runoff proxy[65]. **C.** Chinese cave $\delta^{18}O$ records[66]. **D-E.** East Asian monsoon precipitation and wind-intensity proxies (carbonate $\delta^{13}C$ and mean grain size) from Chinese Jingyuan (JY) Loess[67]. **F.** Indian Summer Monsoon stack: composite records of grain size, XRF-scanner bromine counts and foraminiferal assemblages in core MD04-286[68]. **G.** Ice volume corrected seawater oxygen isotopes ($\delta^{18}O_{sw}$) from Site U1446 as Bay of Bengal salinity/Indian subcontinent runoff proxy[38]. **H.** Ice volume corrected seawater oxygen isotopes ($\delta^{18}O_{sw}$) from Andaman Sea Site NGHP17 as eastern Bay of Bengal Irrawaddy-Salween river runoff proxy[69].

Supplementary Figure S17 provides a comparison of post-MPT proxy records for the East Asian (runoff/salinity, loess and cave records) and the Indian/South Asian monsoon systems (runoff/salinity and chemical weathering records). With the exception of the Chinese cave records[66] and the Indian Summer Monsoon stack[68], these records exhibit a significant 100 kyr cycle associated with glacial/interglacial climate variability over the past 950 kyr. Precessional signals are strong in all records except for Sites U1429 (East China Sea)[65] and U1467 (Maldives)[64]. Significant obliquity signals are additionally embedded in the Chinese cave and loess records[67] as well as in the Andaman Sea $\delta^{18}O_{sw}$ record at NGHP17[69].

Supplementary References

1. Gong, L., Lübbers, J., Beil, S. & Holbourn, A. Data report: revised composite depth scale and splice for IODP Expedition 363 Site U1483. *In Proc.IODP 363* Vol. **363,** International Ocean Discovery Program, College Station, TX (2020).
2. Lisiecki, L. E. & Raymo, M. E. A Pliocene-Pleistocene stack of 57 globally distributed benthic $\delta^{18}O$ records. *Paleoceanography* **20,** PA1003 (2005).
3. Zhang, P. et al. Indo-Pacific hydroclimate in response to changes of the intertropical convergence zone: Discrepancy on precession and obliquity bands over the last 410 kyr. *J. Geophys. Res. Atmos.* **125,** e2019JD032125 (2020).
4. Holbourn, A. et al. Orbitally paced paleoproductivity variations in the Timor Sea and Indonesian Throughflow variability during the last 460 kyr. *Paleoceanography* **20,** 3002 (2005).
5. Kawamura, H., Holbourn, A. & Kuhnt, W. Climate variability and land–ocean interactions in the Indo Pacific Warm Pool: A 460-ka palynological and organic geochemical Record from the Timor Sea. *Mar. Micropal.* **59,** 1–14 (2006).
6. Ziegler, M., Jilbert, T., de Lange, G. J., Lourens, L. J. & Reichart, G. J. Bromine counts from XRF scanning as an estimate of the marine organic carbon content of sediment cores. *Geochem. Geophys. Geosyst.* **9,** Q05009 (2008).
7. Seki, A., Tada, R., Kurokawa, S. & Murayama, M. High-resolution Quaternary record of marine organic carbon content in the hemipelagic sediments of the Japan Sea from bromine counts measured by XRF core scanner. *Progr. Earth and Planet. Sci.* **6,** 1-12 (2019).

8. Szérémeta, N., Bassinot, F., Balut, Y., Labeyrie, L. & Pagel, M. Oversampling of sedimentary series collected by giant piston corer: Evidence and corrections based on 3.5-kHz chirp profiles, *Paleoceanography* **19,** PA1005 (2004).
9. Cumberland, S. A. et al. Uranium mobility in organic matter-rich sediments: A review of geological and geochemical processes. *Earth. Sci. Rev.* **159,** 160–185 (2016).
10. Calvert, S. E. & Pedersen, T. F. Geochemistry of recent oxic and anoxic marine sediments: implications for the geological record. *Mar. Geol.* **113,** 67–88 (1993).
11. Tribovillard, N. et al. Trace metals as paleoredox and paleoproductivity proxies: an update. *Chem. Geol.* **232,** 12–32 (2006).
12. Klinkhammer, G. P. & Palmer M. R. Uranium in the oceans: where it goes and why. *Geochim. Cosmochim. Acta* **55,** 1799–1806 (1991).
13. Khaustova, N. et al. The Study of Uranium Accumulation in Marine Bottom Sediments: Effect of Redox Conditions at the Time of Sedimentation. *Geosciences* **11,** 332 (2021).
14. Mangini, A., Jung, M. & Laukenmann, S. What do we learn from peaks of uranium and of manganese in deep sea sediments? *Mar. Geol.* **177,** 63–78 (2001).
15. Calvert, S. E. & Pedersen, T. F. Sedimentary geochemistry of manganese: implication for the environment of formation of manganiferous black shales. *Econ. Geol.* **91,** 36–47 (1996).
16. Tjallingii, R., Röhl, U., Kölling, M. & Bickert. T. Influence of the water content on X-ray fluorescence core-scanning measurements in soft marine sediments. *Geochemistry, Geophysics, Geosystems* **8,** Q02004 (2007).
17. Hennekam, R. & de Lange, G. X-ray fluorescence core scanning of wet marine sediments: methods to improve quality and reproducibility of high-resolution paleoenvironmental records. *Limnol. Oceanogr. Methods* **10,** 991–100 (2012).
18. Rosenthal, Y. et al. Western Pacific Warm Pool. *Proc. IODP 363* Vol. **363,** (International Ocean Discovery Program, College Station, TX 2018).
19. De Vleeschouwer, D. Natural Gamma Radiation-derived K, U and Th contents of marine sediments obtained during IODP Expeditions with R/V JOIDES Resolution, Version 1.0. Interdisciplinary Earth Data Alliance (IEDA) https://doi.org/10.1594/IEDA/100668 (2017).
20. Hammer, Ø., Harper, D. A. T., and Ryan, P. D. PAST: Paleontological Statistics Software Package for Education and Data Analysis. *Palaeontol. Electron.* **4,** 9 (2001).

21. Schulz, M. & Mudelsee, M. REDFIT: estimating red-noise spectra directly from unevenly spaced paleoclimatic time series. *Computers & Geosciences* **28,** 421–426 (2002).
22. Pei, R. et al. Monitoring Australian Monsoon variability over the past four glacial cycles. *Palaeogeogr. Palaeoclimatol. Palaeoecol.* **568,** 110280 (2021).
23. Tjallingii, R., Stattegger, K., Wetzel, A. & Van Phach, P. Infilling and flooding of the Mekong River incised valley during deglacial sea-level rise. *Quat. Sci. Rev.* **29,** 1432–1444 (2010).
24. Mohtadi, M. et al. Glacial to Holocene swings of the Australian-Indonesian monsoon. *Nat. Geosci.* **4,** 540–544 (2011).
25. Stuut, J. B. W., Temmesfeld, F. & De Deckker, P. A 550 ka record of aeolian activity near North West Cape, Australia: inferences from grain-size distributions and bulk chemistry of SE Indian Ocean deep-sea sediments. *Quat. Sci. Rev.* **83,** 83–94 (2014).
26. Kuhnt, W. et al. Southern Hemisphere control on Australian monsoon variability during the late deglaciation and Holocene. *Nat. Commun.* **6,** 1–7 (2015).
27. Yarincik, K. M., Murray, R. W. & Peterson L. C. Climatically controlled eolian and hemipelagic deposition in the Cariaco Basin, Venezuela, over the past 578,000 years: Results from Al/Ti and K/Al. *Paleoceanography* **15,** 210–228 (2000).
28. Zabel, M., Schneider, R. R., Wagner, T., Adegbie, A. T., deVries, U & Kolonic, S. Late Quaternary climate changes in Central Africa as inferred from terrigenous input to the Niger Fan. *Quat. Res.* **56,** 207–217 (2001).
29. Clift, P. D., Hodges, K. V., Heslop, D., Hannigan, R., Hoang, L. V. & Calves, G. Correlation of Himalayan exhumation rates and Asian monsoon intensity. *Nat. Geosci.* **1,** 875–880 (2008).
30. Gingele, F. X. & De Deckker, P. Fingerprinting Australia's rivers with clay minerals and the application for the marine record of climate change. *Australian Journal of Earth Sciences* **51,** 339–348 (2004).
31. Gebregiorgis, D. et al. What can we learn from X-ray fluorescence core scanning data? A paleomonsoon case study. *Geochemistry, Geophysics, Geosystems* **21,** e2019GC008414 (2020).
32. Kuhnt, W. et al. Southern Hemisphere control on Australian monsoon variability during the late deglaciation and Holocene. *Nat. Commun.* **6,** 1–7 (2015).

33. Stuut, J. B. W., Temmesfeld, F. & DeDeckker, P. A 550 ka record of aeolian activity near North West Cape, Australia: inferences from grain-size distributions and bulk chemistry of SE Indian Ocean deep-sea sediments. *Quat. Sci. Rev.* **83,** 83–94 (2014).
34. Stuut, J. B. W. et al. A 5.3-million-year history of monsoonal precipitation in northwestern Australia. *Geophys. Res. Lett.* **46,** 6946–6954 (2019).
35. Vasiliev, M. et al. A new natural gamma radiation measurement system for marine sediment and rock analysis. *J. Appl. Geophys*. **75,** 455–463 (2011).
36. Laskar, J. et al. A long-term numerical solution for the insolation quantities of the Earth. *Astron. Astrophys.* **428,** 261–285 (2004).
37. Paillard, D., Labeyrie, L. & Yiou, P. Macintosh program performs time-series analysis. *Eos. Trans. AGU* **77,** 379–379 (1996).
38. Clemens, S. C., Yamamoto, M., Thirumalai, K., Giosan, L., Richey, J. N., Nilsson-Kerr, K., Rosenthal, Y., Anand, P. & McGrath, S. M. Remote and local drivers of Pleistocene South Asian summer monsoon precipitation: A test for future predictions. *Sci. Adv.* **7,** 3848 (2021).
39. Lüthi, D. et al. High-resolution carbon dioxide concentration record 650,000-800,000 years before present. *Nature* **453,** 379–382 (2008).
40. Berends, C. J., De Boer, B. & Van De Wal, R. S. Reconstructing the evolution of ice sheets, sea level, and atmospheric CO_2 during the past 3.6 million years. *Clim. Past* **17,** 361–377 (2021).
41. Monnin, E. et al. Atmospheric CO_2 concentrations over the last glacial termination. *Science* **291,** 112–114 (2001).
42. Petit, J. et al. Climate and atmospheric history of the past 420,000 years from the Vostok ice core, Antarctica. *Nature* **399,** 429–436 (1999).
43. Pepin, L., Raynaud, D., Barnola, J. M. & Loutre, M. F. Hemispheric roles of climate forcings during glacial–interglacial transitions as deduced from the Vostok record and LLN-2D model experiments. *J. Geophys. Res.* **106,** 31885–31892 (2001).
44. Raynaud, D. et al. The record for marine isotopic stage 11. *Nature* **436,** 39–40 (2005).
45. Siegenthaler U. et al. Stable carbon cycle-climate relationship during the Late Pleistocene. *Science* **310,** 1313-1317 (2005).
46. McClymont, E. L., Sosdian, S. M., Rosell-Melé, A. & Rosenthal, Y. Pleistocene sea-surface temperature evolution: Early cooling, delayed glacial intensification, and implications for the mid-Pleistocene climate transition. *Earth Sci. Rev.* **123,** 173–193 (2013).

47. Peterson, L. C. et al. Plio-Pleistocene hemispheric (a) symmetries in the Northern and Southern Hemisphere midlatitudes. *Paleoceanogr. Paleoclimatol.* **35,** e2019PA003720 (2020).
48. Carter, R. M. et al. *Proc. ODP, Init. Repts.* **181,** (Ocean Drilling Program, College Station, TX, 1999).
49. Bostock, H. C., Sutton, Phil J., Williams, M. J. M. & Opdyke, B. N. Reviewing the circulation and mixing of Antarctic Intermediate Water in the South Pacific using evidence from geochemical tracers and Argo float trajectories. *Deep-Sea Res. Part I: Oceanogr. Res. Pap.* **73,** 84-98 (2013).
50. Martínez-Garcia, A., Rosell-Melé, A., McClymont, E. L., Gersonde, R. & Haug, G. H. Subpolar link to the emergence of the modern equatorial Pacific cold tongue. *Science* **328,** 1550–1553 (2010).
51. Barker, S. et al. Strengthening Atlantic Inflow Across the Mid-Pleistocene Transition. *Paleoceanogr. Paleoclimatol.* **36,** e2020PA004200 (2021).
52. Rosell-Melé, A., Eglinton, G., Pflaumann, U. & Sarnthein, M. Atlantic core-top calibration of the U^{K}_{37} index as a sea-surface palaeotemperature indicator. *Geochim. Cosmochim. Acta* **59**, 3099–3107 (1995).
53. McClymont, E. L., Rosell-Melé, A., Haug, G., and Lloyd, J. Expansion of subarctic water masses in the North Atlantic and Pacific oceans and implications for mid-Pleistocene ice sheet growth. *Paleoceanography* **23**, PA4214 (2008).
54. Wang, K. J., Huang, Y., Majaneva, M., Belt, S. T., Liao, S., Novak, J., Kartzinel, T. R., Herbert, T. D., Richter, N. and Cabedo-Sanz, P. Group 2i Isochrysidales produce characteristic alkenones reflecting sea ice distribution. *Nat. Commun.* **12**, 15 (2021).
55. Detlef, H. et al. Sea ice dynamics across the Mid-Pleistocene transition in the Bering Sea. *Nat. Commun.* **9**, 941 (2018).
56. Shackleton, N. J., Berger, A. & Peltier, W. R. An alternative astronomical calibration of the lower Pleistocene timescale based on ODP Site 677. *Earth Environ. Sci. Trans. R. Soc. Edinb.* **81,** 251–261 (1990).
57. Berger, A. & Loutre, M. F. Insolation values for the climate of the last 10 million years. *Quaternary Science Reviews* **10,** 297–317 (1991).

58. Lawrence, K. T., Herbert, T. D., Brown, C. M., Raymo, M. E. & Haywood, A. M. High-amplitude variations in North Atlantic sea surface temperature during the early Pliocene warm period. *Paleoceanography* **24,** PA2218 (2009).
59. Herbert, T. D., Peterson, L. C., Lawrence, K. T., & Liu, Z. Tropical ocean temperatures over the past 3.5 million years. *Science* **328,** 1530–1534 (2010).
60. de Garidel-Thoron, T., Rosenthal, Y., Bassinot, F. & Beaufort, L. Stable sea surface temperatures in the western Pacific warm pool over the past 1.75 million years. *Nature* **433,** 294–298 (2005).
61. Russon, T. et al. Inter-hemispheric asymmetry in the early Pleistocene Pacific warm pool. *Geophys. Res. Lett.* **37,** L11601 (2010).
62. Petrick, B. et al. Glacial Indonesian Throughflow weakening across the Mid-Pleistocene climatic transition. *Sci. Rep.* **9,** 16995 (2019).
63. McClymont, E. L. et al. Pliocene-Pleistocene evolution of sea surface and intermediate water temperatures from the southwest Pacific. *Paleoceanography* **31,** 895–913 (2016).
64. Kunkelova, T. et al. A two million year record of low-latitude aridity linked to continental weathering from the Maldives. *Prog. Earth Planet Sci.* **6,** 21 (2019).
65. Clemens, S. C., et al. Precession-band variance missing from East Asian monsoon runoff. *Nat. Commun.* **9,** 11–12 (2018).
66. Cheng, H. et al. The Asian monsoon over the past 640,000 years and ice age terminations. *Nature* **534,** 640–646 (2016).
67. Sun, Y. et al. Diverse manifestations of the mid-Pleistocene climate transition. *Nat. Commun.* **10,** 1–11 (2019).
68. Caley, T. et al. New Arabian Sea records help decipher orbital timing of Indo-Asian monsoon. *Earth Planet. Sci. Lett.* **308,** 433–444 (2011).
69. Gebregiorgis, D. et al. Southern Hemisphere forcing of South Asian monsoon precipitation over the past ~1 million years. *Nat. Commun.* **9,** 1–8 (2018).
